# Identification of Commensal *Escherichia coli* Genes Involved in Biofilm Resistance to Pathogen Colonization

**DOI:** 10.1371/journal.pone.0061628

**Published:** 2013-05-07

**Authors:** Sandra Da Re, Jaione Valle, Nicolas Charbonnel, Christophe Beloin, Patricia Latour-Lambert, Philippe Faure, Evelyne Turlin, Chantal Le Bouguénec, Geneviève Renauld-Mongénie, Christiane Forestier, Jean-Marc Ghigo

**Affiliations:** 1 Institut Pasteur, Unité de Génétique des Biofilms, Département de Microbiologie, Paris, France; 2 Université d'Auvergne-Clermont 1, Laboratoire de Bactériologie, Clermont-Ferrand, France; 3 Université Pierre et Marie Curie, Equipe Neurophysiologie et Comportement (NPC) - UMR 7102, Paris, France; 4 Institut Pasteur, Unité des Membranes Bactériennes, Département de Microbiologie, Paris, France; 5 Institut Pasteur, Unité de Biologie des Bactéries Pathogènes à Gram Positif, Département de Microbiologie, Paris, France; 6 Sanofi Pasteur, Département Discovery, Marcy l'Etoile, France; University of Helsinki, Finland

## Abstract

Protection provided by host bacterial microbiota against microbial pathogens is a well known but ill-understood property referred to as the barrier effect, or colonization resistance. Despite recent genome-wide analyses of host microbiota and increasing therapeutic interest, molecular analysis of colonization resistance is hampered by the complexity of direct *in vivo* experiments. Here we developed an *in vitro*-to-*in vivo* approach to identification of genes involved in resistance of commensal bacteria to exogenous pathogens. We analyzed genetic responses induced in commensal *Escherichia coli* upon entry of a diarrheagenic enteroaggregative *E. coli* or an unrelated *Klebsiella pneumoniae* pathogen into a biofilm community. We showed that pathogens trigger specific responses in commensal bacteria and we identified genes involved in limiting colonization of incoming pathogens within commensal biofilm. We tested the *in vivo* relevance of our findings by comparing the extent of intestinal colonization by enteroaggregative *E. coli* and *K. pneumoniae* pathogens in mice pre-colonized with *E. coli* wild type commensal strain, or mutants corresponding to identified colonization resistance genes. We demonstrated that the absence of *yiaF* and *bssS* (*yceP*) differentially alters pathogen colonization in the mouse gut. This study therefore identifies previously uncharacterized colonization resistance genes and provides new approaches to unravelling molecular aspects of commensal/pathogen competitive interactions.

## Introduction

The mucosal surface of the intestinal tract is a complex ecosystem composed of gastrointestinal epithelium, immune cells and resident bacterial flora. In this environment, bacteria are either in contact with intestinal surfaces or embedded in host-produced mucus [1]–[3]. Genome-wide analyses performed on intestinal microbiota provided insights into beneficial metabolic activities following establishment of successful commensal or symbiotic relationships with the host [4]–[8]. These studies also showed that the absence of an intact microbiota drastically increases susceptibility to pathogens, underlining the fact that colonization of mucosa and competition with commensal bacterial flora is often the first step in most intestinal infections [8]–[10].

This long-known but ill-understood protection provided by commensals against pathogens is commonly described as being colonization resistance, the barrier effect, bacterial antagonism or bacterial interference [1], [10]–[13]. Several mechanisms have been proposed for explaining colonization resistance, including: direct competition for nutrients; prevention of access to adherence sites; limitation of pathogen proliferation through production of inhibitory substances or conditions; or stimulation of host natural immune defenses [10], [14], [15]. However, the complexity of bacterial interactions in the host and the absence of relevant models has severely hindered identification of molecular details on how commensal bacteria interfere with pathogens [13], [16]. Due to these shortcomings, analysis of competitive bacterial interactions that contribute to restricting pathogen establishment within the intestinal flora has almost exclusively focused on secreted inhibitory substances (colicins, microcins, toxins) produced in liquid or solid medium or brought to light in competition experiments performed many decades ago [13].

Recently, interest in bacterial group behavior drew attention to biofilms, swarms, aggregates and dense bacterial cultures as models for studying competitive and synergistic interactions [17]–[26]. Indeed, considering the biofilm-like structure of vertebrate bacterial flora, controlled biofilm communities could enable direct experimental investigations of some aspects of molecular events leading to pathogen establishment in a multispecies context [16], [27], [28].

Here we have developed an *in vitro*-to-*in vivo* approach to studying colonization resistance. We used dynamic and controlled mixed *in vitro* biofilm models to investigate how populations of commensal *Escherichia coli*, a predominant facultative anaerobe of the intestinal microbiota, are colonized by a pathogenic diarrheagenic enteroaggregative *E. coli* [9], [29], [30]. Gene expression profiling demonstrated that pathogen entry into commensal biofilm triggers specific genetic responses, some of them also induced upon colonization by an unrelated bacterial pathogen, *Klebsiella pneumoniae*. Systematic functional analysis led to identification of genes involved in preventing incoming pathogens from settling and growing within commensal biofilm. Finally, we explored the *in vivo* relevance of a subset of identified colonization resistance genes and demonstrated their implication in control of the commensal/pathogen ratio within the mouse gut environment. This study therefore provides new concepts and methods for investigating molecular responses that take place during colonization resistance and that may constitute an early signature in the infection process.

## Materials and Methods

### Bacterial strains and culture media

Bacterial strains are listed in Table 1. All experiments were performed in 0.4% glucose M63B1 minimal medium at 37°C. Antibiotics were added when required, at the following concentrations: ampicillin (100 µg ml^−1^), apramycin (30 µg ml^−1^), tetracycline (7.5 µg ml^−1^), kanamycin (50 µg ml^−1^) and streptomycin (100 µg ml^−1^).

**Table 1:** Strains used in this study.

Strain	Relevant characteristics	References
MG1655	λ*-*, *rph-1*	Laboratory collection
MG1655 F′( = C *in vitro*)	MG1655 carrying the F′*tet*-Δ*traD::apra* plasmid; Apra^R^, Tet^R^	Laboratory collection
55989a ′( = P *in vitro*)	*E. coli* 55989 *λattampgfp*: 55989 with amp*gfp* insertion at the *λ*att site. Amp^R^	(6)
55989a-s( = P *in vivo*)	Spontaneous streptomycin-resistant mutant of *E. coli* 55989a; Amp^R^ Strep^R^.	This study
KpLM21( = P *in vitro*)	*K. pneumoniae* clinical isolate; serogroup O25; Amp^R^	This study
KpLM21-s ( = P *in vivo*)	Spontaneous streptomycin-resistant mutant of *Kp*LM21; Amp^R^, Strep^R^	This study
MG1655*agaI* F′	*ΔagaI*::*GB*, Km^R^, Apra^R^, Tet^R^	This study
MG1655*cspF* F′	*ΔcspF*::*GB*, Km^R^, Apra^R^, Tet^R^	This study
MG1655*kduI* F′	*ΔkduI*::*GB*, Km^R^, Apra^R^, Tet^R^	This study
MG1655*rcsA* F′	*ΔrcsA*::*GB*, Km^R^, Apra^R^, Tet^R^	This study
MG1655*relF* F′	*ΔrelF*::*GB*, Km^R^, Apra^R^, Tet^R^	This study
MG1655*rzpD* F′	*ΔrzpD*::*GB*, Km^R^, Apra^R,^ Tet^R^	This study
MG1655*sppA* F′	*ΔsppA*::*GB*, Km^R^, Apra^R^, Tet^R^	This study
MG1655*stfE* F′	*ΔstfE*::*GB*, Km^R^, Apra^R^, Tet^R^	This study
MG1655*yaeT* F′	*ΔyaeT*::*GB*, Km^R^, Apra^R^, Tet^R^	This study
MG1655*yafX* F′	*ΔyafX*::*GB*, Km^R^, Apra^R^,Tet^R^	This study
MG1655*ycbQ* F′	*ΔycbQ*::*GB*, Km^R^, Apra^R^, Tet^R^	This study
MG1655*yceP* F′	*ΔyceP*::*GB*, Km^R^, Apra^R^,Tet^R^	This study
MG1655*yciF* F′	*ΔyciF*::*GB*, Km^R^, Apra^R^, Tet^R^	This study
MG1655*ydfZ* F′	*ΔydfZ*::*GB*, Km^R^, Apra^R^, Tet^R^	This study
MG1655*yiaF* F′	*ΔyiaF*::*GB*, Km^R^, Apra^R,^ Tet^R^	This study
MG1655*yiaV* F′	*ΔyiaV*::*GB*, Km^R^, Apra^R^, Tet^R^	This study
MG1655*yjcR* F′	*ΔyjcR*::*GB*, Km^R^, Apra^R^, Tet^R^	This study
MG1655*yjiY* F′	*ΔyjiY*::*GB*, Km^R^, Apra^R^,Tet^R^	This study
MG1655*ylcE* F′	*ΔylcE*::*GB*, Km^R^, Apra^R^, Tet^R^	This study
MG1655*yliE* F′	*ΔyliE*::*GB*, Km^R^, Apra^R^, Tet^R^	This study
MG1655*yliH* F′	*ΔyliH*::*GB*, Km^R^, Apra^R^, Tet^R^	This study
MG1655*ypjC* F′	*ΔypjC*::*GB*, Km^R^, Apra^R^, Tet^R^	This study
MG1655PcLrbs-*rcsA* F′	Constitutive expression of *rcsA* from the Km*-*PcLrb*s* cassette, Km^R^	This study
MG1655PcLrbs-*stfE* F′	Constitutive expression of *stfE* from the Km*-*PcLrb*s* cassette, Km^R^	This study
MG1655PcLrbs-*yiaF* F′	Constitutive expression of *yiaF* from the Km*-*PcLrb*s* cassette, Km^R^	This study
MG1655PcLrbs-*yliE* F′	Constitutive expression of *yliE* from the Km*-*PcLrb*s* cassette, Km^R^	This study
MG1655PcLrbs-*ypjC* F′	Constitutive expression of *ypjC* from the Km*-*PcLrb*s* cassette, Km^R^	This study
55989a*-yiaF*	*ΔyiaV*::*GB*, Amp^R^, Km^R^	This study
55989a *-rcsA*	*ΔrcsA*::*GB*, Amp^R^, Km^R^	This study
55989a *-yliE*	*ΔyliE*::*GB*, Amp^R^, Km^R^	This study
MG1655-s F′( = C *in vitro*)	A streptomycin derivative of MG1655 F′, Strep^R^	This study
MG1655-s *yceP* F′	*ΔyceP*::*GB*, Km^R^, Apra^R^, Tet^R^, Strep^R^	This study
MG1655-s *yliE* F′	*ΔyliE*::*GB*, Km^R^, Apra^R^, Tet^R^, Strep^R^	This study
MG1655-s *yiaF* F′	*ΔyiaF*::*GB*, Km^R^, Apra^R,^ Tet^R^, Strep^R^	This study

### Monospecific and mixed biofilm

#### Microfermentor experiments

Biofilms were produced in a continuous flow biofilm microfermentor at 37°C in minimal M63B1 medium supplemented with 0.4% glucose as in (www.pasteur.fr/recherche/unites/Ggb/biofilmfermenter.html) and [31]. Microfermentor inoculations were performed by placing the microfermentor internal spatula in a culture containing 2.10^8^ bacteria/ml for 2 min. The glass slide was then briefly rinsed in minimal media and reintroduced into the microfermentor.

#### Biofilm colonization

After 6 h of continuous culture, biofilm formed on a microfermentor glass slide was re-inoculated by direct introduction of 10^9^ bacteria of overnight cultures of *E. coli* MG1655 F′, *E. coli* 55989a or *K. pneumoniae* KpLM21 bacteria into the microfermentor. Mixed biofilm continuous flow culture was resumed for an additional 24 h (30 h total) with rapid dilution and evacuation of excess planktonic bacteria. For monospecies biofilms, no re-inoculation was performed. Mono- or mixed biofilms formed on the internal microfermentor glass slide were resuspended by vortexing and biofilm biomass was estimated by determining optical density at 600 nm (OD_600 nm_).

#### Colonization phenotype

To estimate the percentage of colonizing bacteria in mixed biofilms, serial dilutions of resuspended biofilm were plated onto LB (total count estimation) and LB with specific antibiotics, thus distinguishing commensal from colonizing exogenous bacteria. All experiments were repeated at least 6 times. Statistical significance of differences observed between colonization phenotypes was estimated by Student t-tests. Differences were considered statistically significant when p<0.05.

### Macroarrays

Genomic expression profiles were performed on *E. coli* MG1655 F′ (C) and 55989a (P) grown as 24 h mono- or mixed biofilms. The equivalent of 15 OD_600 nm_ of bacterial cells were collected, pelleted and rapidly frozen. Cells were then broken in a Fast Prep apparatus (Bio 101) and total RNA was extracted by Trizol (Gibco BRL) treatment. Genomic DNA was removed using RNase-free DNAse I (Roche Diagnostics). Radioactively labeled cDNAs, generated using *E. coli* K-12 CDS-specific primers (Sigma-GenoSys), were hybridized to *E. coli* K-12 panorama gene arrays containing duplicated spots for each of the 4,290 predicted *E. coli* K-12 open reading frames (ORFs; Sigma-GenoSys). The intensity of each dot was quantified with ArrayVision™ software (Imaging Research, Inc.). Experiments were carried out using three independent RNA preparations for each sample condition (C; C+C; C+P; P). Each hybridization with each independent sample was carried out with 1 µg and 10 µg of total RNA; 3 sets of arrays were used.

### Statistical analysis of macroarray data

Genes that were statistically significantly over- or underexpressed were identified using T-test analysis followed by the non-parametric Wilcoxon rank sum test. For each gene, expression in monospecies MG1655 F′ or 55989a biofilm and in self-infected MG1655 F′ + MG1655 F′ or mixed MG1655 F′ + 55989a biofilms (n = 10 to 12 for each data set) were compared. Analyses were performed with one-tailed tests. Genes were considered statistically significantly over- or underexpressed when p<0.05. Low (less than 0.01) or negative levels of expression were removed from the analysis.

### Molecular techniques and construction of deletion and expression mutants

The genome of *E. coli* 55989 was sequenced and annotated by the Coliscope Consortium at the end of the experimental work [32]. *E. coli* 55989 Sequence is deposited in GenBank (accession number NC_011748.1 and GI:218693476). Deletion mutants and introduction of constitutive promoter cassettes in front of described target genes were performed as described at (http://www.pasteur.fr/recherche/unites/Ggb/matmet.html) and in [33], [34] using primers presented in Table S6. DNA sequencing was performed using Eurofins MWG services.

### RT-PCR in *E. coli -K. pneumoniae* mixed biofilms

Biofilm bacteria were directly resuspended in an equal volume of ice-cold RNA*later* (Ambion). Total RNA was isolated and purified using an RNeasy mini-kit (Qiagen). After purification, RNA was treated with RNase-free DNase I to remove contaminating DNA and re-purified using Qiagen RNeasy columns. RNA samples were quantified spectrophotometrically at 260 nm and additionally checked by gel electrophoresis. Purified total RNA was precipitated with ethanol and stored at −80°C until further use. RNA was converted to cDNA using SuperScript II as described by the manufacturer (Invitrogen Life Technologies). cDNA was used directly as template for PCR using specific primers (Table S6). A negative control using the original RNA was consistently run in parallel to confirm the absence of contaminating DNA.

### Mouse model of intestinal colonization

Female IOPS mice (Charles River Laboratories, OF1, 8 to 18 weeks old, 25 g) were used. They were given sterile water containing 5 g/L of streptomycin sulfate throughout the experiment and had *ad libitum* access to feed. After 24 h, 200 µl bacterial suspensions containing 10^6^ cfu of either MG1655-s F′ or its mutants (MG1655-s *yliE* F′, MG1655-s *yceP* F′, MG1655-s *yiaF* F′) were given intragastrically. At least eight mice were infected with the wild-type strain and another eight mice with each mutant strain. At day 11, each animal was administrated intragastrically 10^2^ cfu of the pathogenic strain, 55989a-s or 10^3^ cfu KpLM21-s. These doses correspond to minimal inocula to detect pathogen colonization in feces. On day 3, and subsequently every other day after inoculation, feces were collected, homogenized in 0.9% saline, and serial dilutions were plated onto both tetracycline-containing media (detection of MG1655-s F′) and ampicillin-containing media (detection of the pathogen). The potential impact of initial colonization by wild-type MG1655-s F′ or its derivatives upon the capacity of the pathogens (EAEC 55989 or *K. pneumoniae*) to colonize was assessed by calculating the Pearson correlation coefficient using the number of cfus determined at days 10 and 12. A Mann-Whitney statistical test was then used to assess the colonization capacity of each pathogen (*K. pneumoniae* and EAEC) by comparing the number of cfus from D12 to D20 in feces of mice previously inoculated with the *yliE*, *yceP* or *yiaF* mutant to the number of pathogens observed in mice previously colonized with wild-type MG1655-s F′. A *P* value of <0.05 was considered statistically significant.

### Ethics statement

Animal studies were performed in accordance with the European Community guiding in the care and use of animals (86/609/CEE). Furthermore, the models and protocols used in this study were all approved by the ethics committee of Auvergne (Comité Régional d'Ethique en Matière d'Expérimentation Animale Auvergne). Animals were housed under controlled environmental conditions and kept under a 12/12 h light/dark cycle, with food and water *ad libitum*.

## Results

### A new in vitro model of commensal biofilm colonization by exogenous pathogens

To identify the genetic responses triggered in a commensal biofilm upon entry of exogenous pathogens, we developed an *in vitro* model in which pathogenic bacteria were exogenously added to an already formed commensal biofilm. This procedure will be referred to as *biofilm colonization* throughout this study. As a biofilm-forming commensal bacterium (or C for commensal), we chose *E. coli* K12 MG1655 F′ carrying a conjugation-deficient derivative of the F conjugative plasmid (F′tet*ΔtraD*) that rapidly forms biofilm under continuous flow microfermentor conditions [31]. The pathogenic strain (P) chosen to colonize MG1655 F′ commensal biofilm is an ampicillin-resistant derivative of *E. coli* 55989, a biofilm-forming enteroaggregative (EAEC) isolate originally isolated from diarrheagenic stools and causing acute and persistent diarrhea [9], [35], hereafter referred to as 55989a or P.

**Figure 1:**
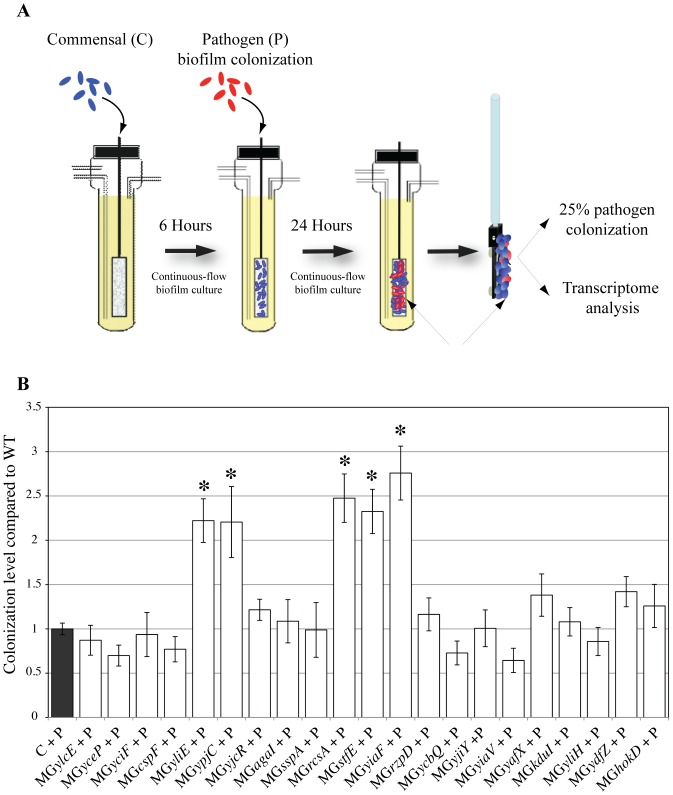
Identification of colonization resistance factors interfering with establishment of mixed pathogen/commensal biofilm. **A** Experimental set-up: continuous flow biofilm growth in microfermentor. After initial inoculation of the microfermentor with *E. coli* MG1655 F′ commensal (C), biofilm develops for 6 h before re-inoculation (colonization) with exogenous pathogen *E. coli* 55989a (P). At 24 h post-colonization, mixed biofilm developing on the glass slide was resuspended and used for gene expression analysis and determination of colonization phenotype (% of pathogens in the mixed biofilm). **B** Microfermentors were inoculated with wild-type or mutant commensal (MG1655 F′ is abbreviated as MG) as indicated in the x-axis. After 6 h of growth, commensal biofilm was re-inoculated (colonized) with the 55989a (P). Colonization phenotype of each mixed biofilm was estimated and results are represented as ratio of colonization level in C*mutant*+P mixed biofilms compared to wild-type C+P mixed biofilms. Black bar represents wild-type colonization level in C+P mixed biofilms arbitrarily set to one. White bars represent colonization level of C*mutant*+P mixed biofilms. Results are averages of at least 6 replicates ± standard deviation of the mean. Stars indicate mutant mixed biofilm with a colonization level significantly different from that of wild-type C+P mixed biofilm, p<0.01.

To establish conditions of MG1655 F′ colonization upon exogenous introduction of 55989a, we first produced MG1655 F′ biofilms formed for 6 to 24 h in continuous flow microfermentors. We then inoculated them with various titers of *E. coli* 55989a and allowed the resulting mixed biofilm to grow an additional 24 h. We defined *E. coli* 55989a colonization efficiency as the percentage of pathogens present in the resulting 24 h C+P mixed biofilm, as determined using the 55989a ampicillin antibiotic resistance marker (Table 1). At 24 h, a commensal colony-forming unit (cfu) had increased by a 2-log factor and the presence of the pathogen did not significantly alter development of the commensal biofilm, since C and C+P biofilm displayed similar biomass (data not shown). We found that the proportion of 55989a in C+P biofilm depended on both the 55989a initial inoculation titer and the age of MG1655 F′ biofilm. When MG1655 F′ 6 h biofilms were inoculated with a titer of 10^9^ bacteria/ml of 55989a, we reproducibly obtained 25+/−5% of 55989a in 24 h C+P mixed biofilm; we used these experimental conditions throughout the rest of the study (Fig. 1A).

### Pathogen colonization of commensal biofilm triggers specific genetic responses

To investigate the genetic response of MG1655 F′ biofilm bacteria upon introduction of *E. coli* 55989a, we first compared the expression profile of monospecific MG1655 F′ biofilm (C) to that of both monospecific 55989a biofilm (P) and “self-mixed” biofilm, in which MG1655 F′ commensal biofilm was colonized alone (C+C). Comparison of monospecific biofilm (P/C), indicated that, among genes common to both strains, 545 exhibited differing expression profiles (Table 2). Analysis of “self-mixed” or self-colonization, abbreviated as (C+C/C), showed that 346 genes underwent significant transcription level changes between the two conditions, indicating that addition of an exogenous but identical commensal bacterium to commensal biofilm already induces changes in gene expression (see Tables 2, S2 and S3). We then compared bacterial gene expression in mixed MG1655 F′+55989a (C+P) biofilm with gene expression in monospecies commensal MG1655 F′ (C) biofilm. This analysis, abbreviated as (C+P/C), revealed the differential expression of 329 genes between the two biofilms (see Tables 2, S4 and S5).

**Table 2:** Summary of transcriptome analyses performed on biofilms colonized by different exogenous bacteria.

Conditions	Number of genes repressed or overexpressed^e^			Functional categories (COG) (% of total number)			
		Total	≥2 fold	Information storage and processing	Cellular processes	Metabolism	Unknown^f^
P/C^a^	Repressed	389	163	22.4	21.3	21.1	35.2
	Overexpressed	156	18	16.7	19.9	28.2	35.2
	All	545					
C+C/C^b^	Repressed	185	60	14.1	12.4	19.5	54
	Overexpressed	161	2	14.3	21.7	42.2	21.8
	All	346					
C+P/C^c^	Repressed	109	4	14.7	18.3	22.9	44
	Overexpressed	220	4	12.7	23.2	38.2	25.9
	All	329					
C+P/C+C^d^	Repressed	61	1	21.3	9.8	26.2	42.6
	Overexpressed	108	32	15.7	16.7	33.3	34.3
	All	169					

a : Monospecies pathogen *E. coli* 55989a biofilm (P) versus monospecies commensal *E. coli* K12 MG1655 F′ (C): comparison.

b : Commensal biofilm infected by identical commensal bacteria (C+C) versus monospecies commensal biofilm (C): comparison.

c : Commensal biofilm infected by *E. coli* 55989a (mixed biofilm, C+P) versus monospecies commensal biofilm (C): comparison.

d : Commensal biofilm infected by *E. coli* 55989a (mixed biofilm, C+P) versus commensal biofilm infected by identical commensal bacteria (C+C): comparison.

e : p<0.05.

f : “Unknown” regroups poorly characterized and unknown function genes of the COG classification plus non-classified genes coding mainly for hypothetical proteins.

Comparison of gene expression upon self-colonization (C+C/C experiment) and upon pathogen colonization (C+P/C experiment) showed a common genetic response to entry of exogenous bacteria into commensal MG1655 F′ biofilm, with the same 89 overexpressed and 26 repressed genes in both situations (see Tables S4 and S5). Moreover, comparison of non-self versus self-colonized analyses (C+P/C+C comparison) indicated significant specific differential gene expression in response to 55989a colonization, with 61 repressed genes and 108 overexpressed genes. The distribution of these 169 genes in the different COG functional classes is comparable to that found in C+P/C transcription profile analysis, including 30 to 40% of poorly characterized genes (Tables 2, S4 and S5). Furthermore, several overexpressed and underexpressed genes overlapped C+P/C and C+P/C+C analysis, (Table S1), suggesting that these genes could be involved in specific responses to colonization of *E. coli* commensal biofilm by pathogenic bacteria.

### Colonization responses of commensal biofilm bacteria reduce pathogen colonization

Although our analysis focused on *E. coli* MG1655 genes, most of these genes are also present on the *E. coli* 55989 genome and we could not rule out that the observed variation in transcription also reflected responses induced in the 55989a pathogen. We nevertheless hypothesized that the identified genetic responses could contribute to limiting pathogen colonization within MG1655 F′ commensal biofilm. To test this, we selected 22 genes differentially expressed by MG1655 F′ biofilm bacteria upon colonization by 55989a, by comparing the C+P/C and C+P/C+C versus C+C/C datasets. These 22 genes included genes coding for membrane and envelope proteins (36%), proteins of phage origin (27%), regulators implicated in biofilm-associated phenotype (13%) and genes of unknown function (14%), (Table 3 and Fig. S1). RT-PCR experiments on six of the most frequently expressed of these 22 genes (*hokD*, *kduI*, *ylcE*, *yceP*, *yliH* and *yciF*) indicated that, although ratios obtained with the two approaches differed, all tested genes displayed increased levels of transcription within mixed C+P biofilm compared to mixed C+C biofilm (data not shown). We then performed non-polar deletion of the 22 selected genes in commensal strain MG1655 F′. With the exception of *yaeT (bamA*), all mutants exhibited wild-type growth and biofilm formation ability (data not shown). Biofilms corresponding to the 21 remaining mutants were inoculated with wild-type 55989a using the procedure described in Fig. 1A. Determination of the percentage of pathogens in resulting mixed C+P biofilms showed that biofilms formed by MG1655 F′ mutants *yliE*, *ypjC*, *rcsA*, *stfE* and *yiaF* mutant were colonized by the pathogen 55989a at significantly higher levels than the wild-type strain (p<0.01) (Fig. 1B). *yliE* encodes a conserved inner membrane hypothetical protein of 90 kDa that contains an EAL domain characteristic of phosphodiesterase enzymes degrading c-di-GMP and often involved in transition between individual and community lifestyles [36]; *ypjC* encodes a hypothetical protein of 18 kDa; *rcsA* codes for a cofactor required for synthesis of colanic acid, capsular polysaccharide and curli synthesis in *E. coli* [37], [38]; *stfE* encodes a putative tail protein homolog from lambdoid prophage e14 and *yiaF* encodes a 25.6 kDa inner membrane protein exhibiting patchy distribution with polar and septal bias [39].

**Table 3:** Selection of genes differentially expressed in C+P/C and C+P/C+C, but not in C+C/C analysis.

Gene	Gene function	Macroarray analysis (ratio)^a^		
		C+P/C	C+P/C+C	C+C/C
*hokD* *	(*relF*); cell killing	4.85	7.52	0.4
*kduI*	Homolog of pectin-degrading enzyme 5-keto 4-deoxyuronate isomerase	2.88	2.56	
*ylcE* *	(*tfaX*); tail fiber assembly predicted protein, DLP12 prophage		2.55	
*yceP*	(*bssS*) biofilm regulator through signal secretion		2.52	0.51
*yliH*	(*bssR*) putative receptor, biofilm regulator through signal secretion	2.47	2.15	
*yciF*	Putative structural protein		2.01	0.61
*cspF* *	Cold shock protein homolog		1.99	
*ypjC*	Hypothetical protein		1.99	
*yliE*	Predicted cyclic-di-GMP phosphodiesterase, inner membrane protein	1.99		
*sppA*	Protease IV, signal peptide peptidase	1.90	1.92	
*agaI*	6-Phosphogluconolactonase/glucosamine-6-phosphate isomerase/deaminase	1.89	2.37	
*rcsA*	Positive regulatory gene for capsule (colanic acid) synthesis		1.88	
*ydfZ*	Hypothetical protein, potential seleno-protein	1.79	2.77	0.65
*yjcR*	(*sdsR*); putative membrane protein of multidrug efflux pump	1.74		
*stfE* *	Putative tail fiber protein, e14 prophage		1.71	
*yiaF*	Inner membrane protein	1.65		
*rzpD* *	Endopeptidase-like protein, DLP12 prpophage	1.62		
*yaeT*	(*bamA*); integral β-barrel protein, outer membrane	1.58		
*ycbQ*	Chaperone-usher fimbrial protein	1.57		
*yafX* *	Hypothetical protein, CP4-6 prophage	1.55		
*yjiY*	Inner membrane protein; putative carbon starvation protein	1.53		
*yiaV*	Inner membrane protein, predicted component of efflux pump	1.50		

a Compiled from Tables S2 and S4. No value given when gene expression did not change at a statistically significant level in the analysis in question.

* Protein related to prophage.

The increased pathogen colonization phenotype obtained for the 5 mutants correlated with a slight decrease in commensal bacteria cfu in mixed C+P biofilm (1.5-fold average for the *rcsA*, *stfE*, *yliE* and *ypjC* mutants and 2.3-fold for the *yiaF* mutant), associated with a 2-fold increase in pathogen cfu. However, estimation of bacterial cfu constituting the biofilm of MG1655 F′ *yiaF*, *stfE* and *yliE* mutants at the time of infection (6H) showed no significant difference from wild-type MG1655 F′, indicating that the increased 55989a pathogen colonization phenotype obtained with these mutants was not due to decreased ability to form the initial biofilm (Fig. S2). In contrast, MG1655*rcsA* F′ mutant biofilm biomass was reduced, while the number of bacteria recovered from MG1655*ypjC* F′ biofilms was 3.5-fold higher than in wild-type MG1655 F′ commensal biofilm (Fig. S2).

**Figure 2:**
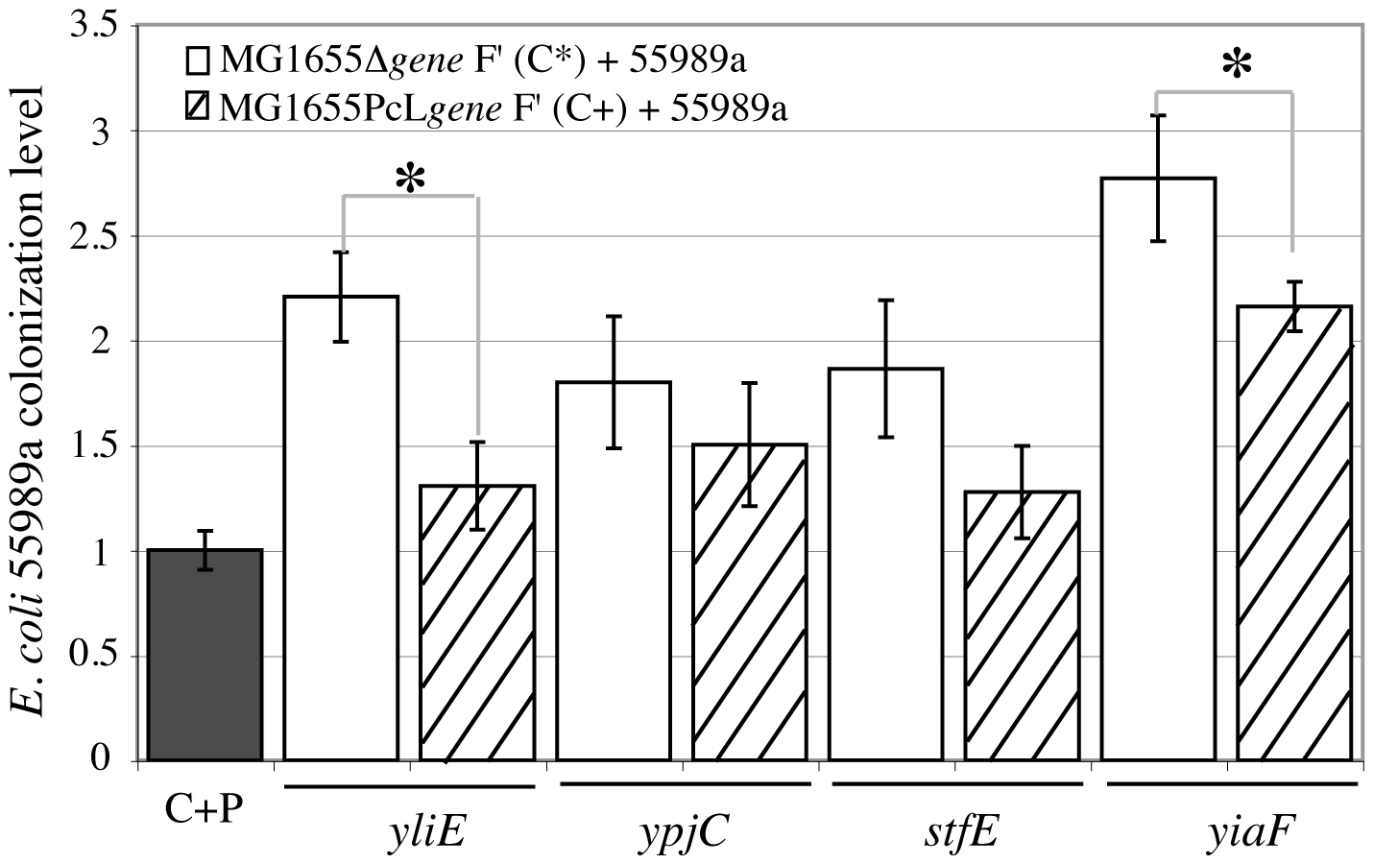
Constitutive expression of potential colonization resistance genes. Estimate of *E. coli* 55989a (P) colonization in mixed biofilms with wild-type *E. coli* MG1655 F′ (C), corresponding deletion mutants (MG1655Δ*gene*: C*) or overexpressed (MG1655PcL*gene:* C+) derivative strains. Results are represented as ratio of colonization l of mutant mixed biofilms to wild-type mixed C+P biofilms. Black bar represents wild-type colonization in C+P mixed biofilms arbitrarily set to one. White bars represent colonization of pathogen in mixed C*Δgene* + P biofilms. Stripped bars show pathogen colonization in mixed C*PcLgene* + P biofilms with commensal overexpressing potential colonization resistance genes. Genes deleted or overexpressed are indicated under the bars. Results are averages of at least 12 replicates ± standard deviation of the mean. The extent of colonization in C*Δgene* + P mixed biofilm was significantly different from that of wild-type C+P biofilm *p<*0.05; asterisks indicate significant difference between extent of colonization in over-expressed and deletion mutants, *p<*0.05.

We then used a previously described plasmid-free expression strategy and placed a constitutive promoter in front of the *yiaF*, *stfE*, *yliE* and *ypjC* genes directly on the MG1655 F′ chromosome [34], [40]. This insertion had no effect on growth or biofilm formation for *yiaF*, *stfE*, *yliE* and *ypjC* mutant strains (data not shown). However, constitutive expression of *rcsA* (MG1655PcLrbs-*rcsA* F′) led to mucoidy and the inability to form biofilm (data not shown), and this mutant was not further analyzed. The colonization phenotype of the 4 remaining mutants was evaluated and compared to that of the corresponding deletion mutants. While constitutive expression of *ypjC* and *stfE* did not lead to significant 55989a colonization changes, *yiaF* and *yliE* constitutive expression in MG1655PcLrbs-*yiaF* F′ and MG1655PcLrbs-*yliE* F′, respectively, significantly reduced the ability of *E. coli* pathogen 55989a to colonize the resulting C+P mixed biofilm compared to colonization of the corresponding MG1655 F′ deletion mutants (Fig. 2).

**Figure 3:**
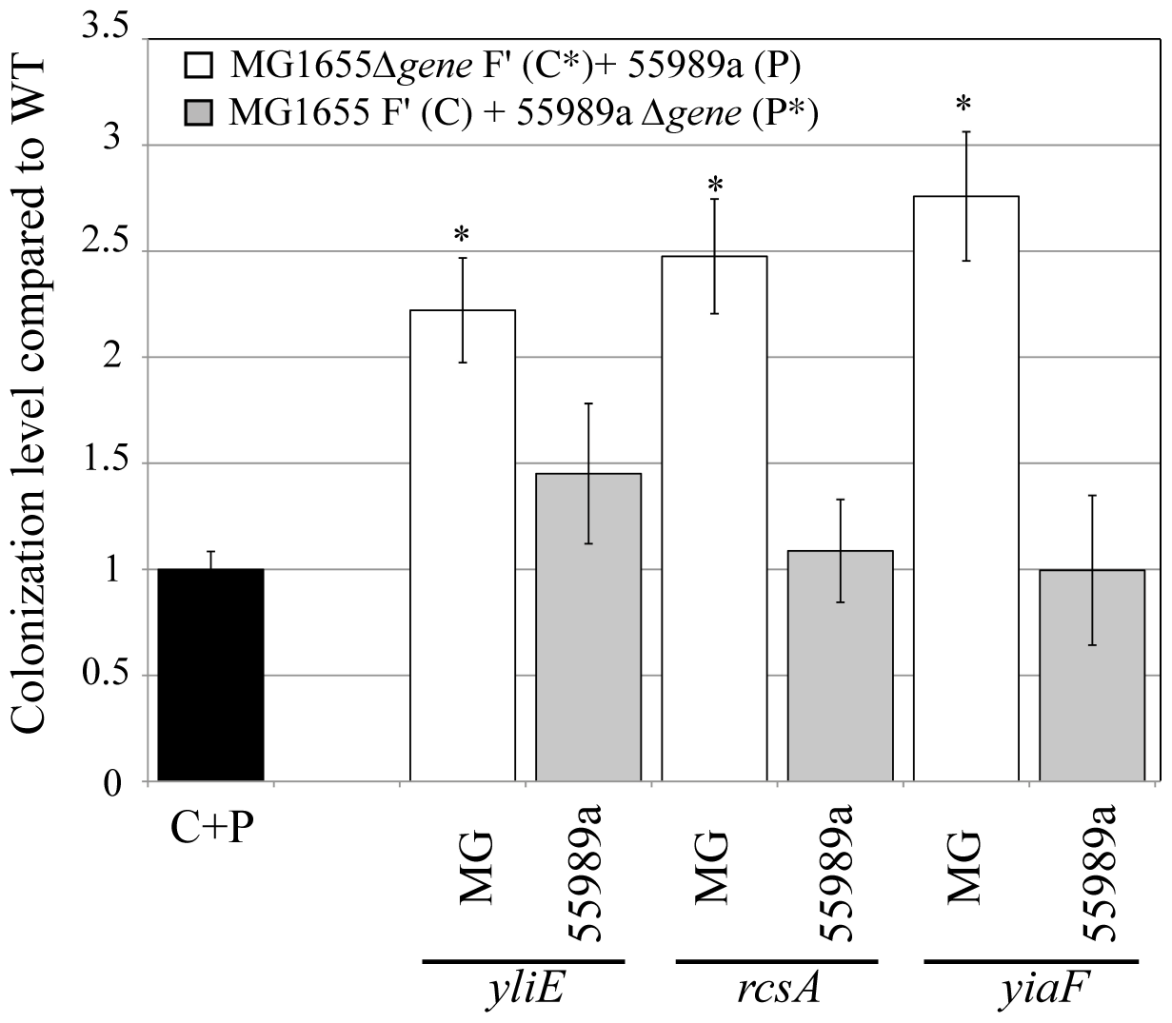
Colonization resistance genes are strain-specific. Comparison of the effect on colonization of mutations introduced into commensal MG1655 F′ (C) or into pathogenic strain 55989a (P). Results are represented as ratio of colonization of mutant mixed MG1655 F′Δ*gene* (C*) +P or C+55989aΔ*gene* (P*) biofilms compared to wild-type mixed C+P biofilm. Black bar represents extent of wild-type colonization in C+P mixed biofilms arbitrarily set to one. White bars represent colonization levels of CΔ*gene* +P mixed biofilm (mutation introduced into commensal and wild-type pathogens). Light gray bars represent colonization levels of C+PΔ*gene* mixed biofilm formed by wild-type commensal and mutant pathogens. Names of deleted genes are indicated under the line. Results are averages of at least 6 replicates ± standard deviation of the mean. Asterisks indicate mutant mixed biofilm with a colonization level significantly different from that of wild-type MG1655 F′ + 55989a mixed C+P biofilm, *P<*0.05.

Finally, we tested whether *yiaF*, *stfE*, *rcsA*, *yliE* and *ypjC* could also play a reciprocal role in *E. coli* 55989a ability to colonize a commensal biofilm. While *stfE* was absent from the 55989 genome, a *ypjC* mutant could not be obtained despite repeated attempts. We therefore introduced only a *yiaF*, *rcsA* or *yliE* mutation in the 55989a strain, and we showed that none of these 3 mutations had a significant effect on colonization outcome, suggesting that the observed colonization phenotypes specifically affected pathogen colonization in commensal biofilm, but not the reverse (Fig. 3). Taken together, these results indicated that colonization of commensal MG1655 F′ biofilm by the diarrheagenic pathogenic strain 55989a triggers expression of commensal genes contributing to colonization resistance to the pathogen.

### Common genetic responses of commensal biofilm upon colonization by distinct pathogens

We investigated whether *in vitro* commensal biofilm colonization by a bacterium other than *E. coli* 55989a could trigger similar genetic responses. We inoculated a pre-formed MG1655 F′ commensal biofilm with the biofilm-forming opportunistic enterobacterium *Klebsiella pneumoniae* strain LM 21 (hereafter referred to as KpLM21) responsible for a wide range of nosocomial infections, including pneumonia, bacteremia and urinary tract infections [41], [42]. *K. pneumoniae* can also be found in the intestine, where it can colonize the local microbiota. Conditions similar to those used with EAEC strain 55989a resulted in mixed biofilm composed of 75% MG1655 F′ and 25% KpLM21. We then monitored expression of 6 genes absent from the *K. pneumoniae* genome and expressed in MG1655 F′ in response to *E. coli* 55989a colonization. *yiaF*, *yliE* and *stfE* were chosen for their contribution to MG1655 F′ colonization resistance against 55989a, while *yceP* and *yliH* (*bssR* and *bssS*, respectively, for “regulator of biofilm through signal secretion”, see Discussion) were selected because they are overexpressed in mixed C+P biofilm compared to uninfected and/or self-infected biofilm (Table 3) [43], [44]. RT-PCR on RNA extracted from mixed 24 h C+P (*E. coli + K. pneumoniae*) biofilms showed that, although colonization of commensal MG1655 F′ biofilm by KpLM21 had no impact on *stfE* expression and reduced *yliE* expression, it led to induction of *yiaF*, *yceP* and *yliH* (Table 4 and Fig. S1)

**Table 4:** Gene expression level in mixed MG1655F′ + *K. pneumoniae* biofilms.

Gene	Fold induction^a^	T-test
*yiaF*	1.62±0.13	0.034
*yliE*	0.72±0.04	0.043
*stfE*	0.83±0.09	0.902
*yliH*	1.41±0.11	0.047
*yceP*	1.69±0.20	0.037

a Gene expression level was estimated by RT-PCR in single MG1655 F′ biofilm and mixed MG1655F′ + *K. pneumoniae* KpLM21 (MG+Kp). Gene expression level in MG+Kp biofilm was compared to gene expression in commensal biofilm set to 1. Results are averages of 3 replicates with triplicate measurements for each ± standard deviation of the mean.

These observations indicated the existence of common genetic responses upon colonization of *E. coli* commensal biofilm by two different exogenous bacterial pathogens.

### Correlation between in vitro and in vivo reduction of pathogen colonization

**Figure 4:**
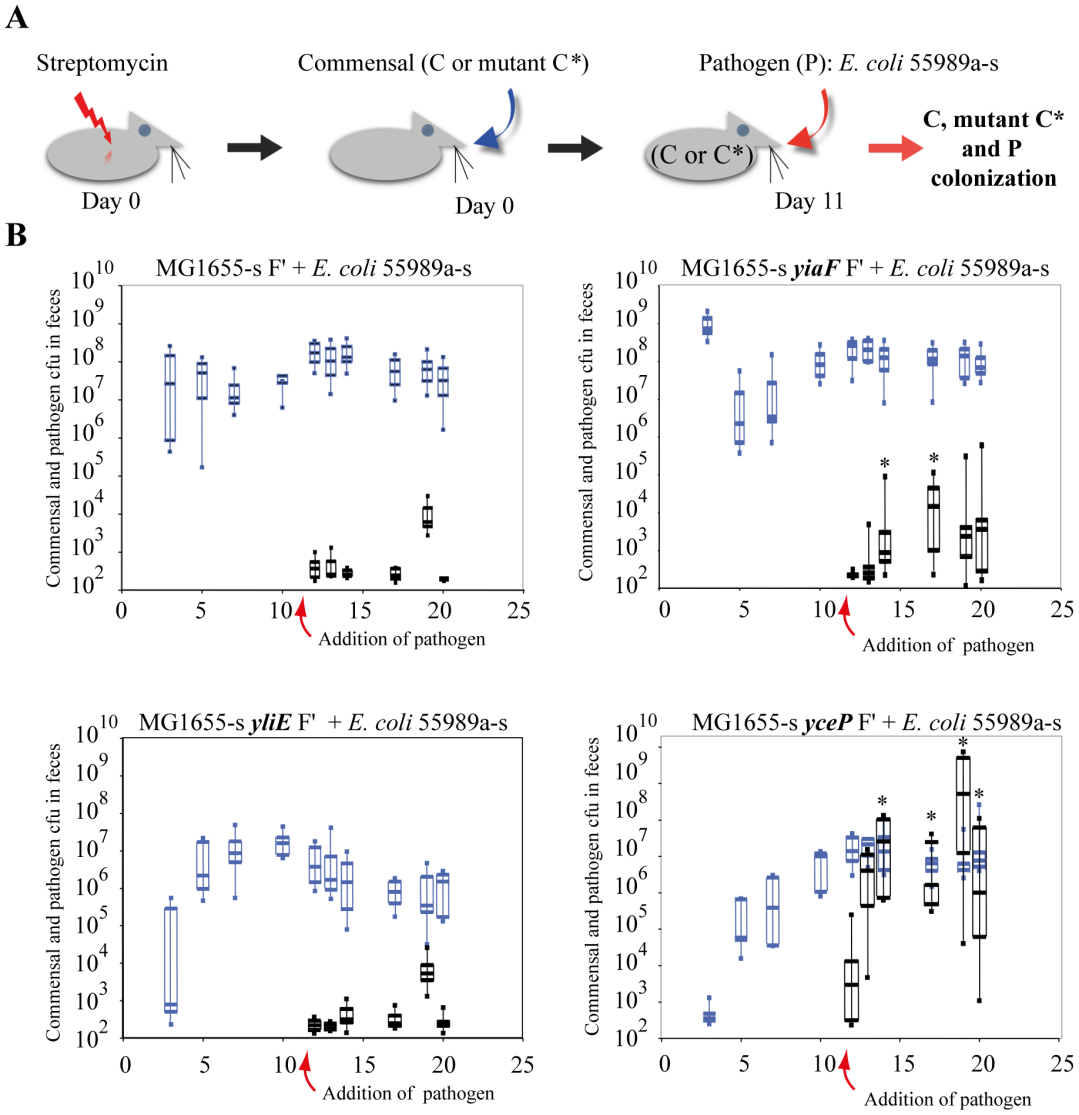
*In vivo* colonization of *E. coli* commensal biofilm by enteroaggregative *E. coli* 55989 pathogen. **A** Schematic representation of the experimental procedure. **B** Streptomycin-treated mice were first challenged intragastrically with commensal wild-type MG1655-s F′ (C) or its mutant Δ*yceP*, Δ*yliE*, and Δ*yiaF* derivatives (C*), followed on day 11 by administration of the *E. coli* 55989a-s pathogen. Numbers of commensal and pathogenic cfus recovered per gram of feces were determined every other day from day 3 to day 20. The lower limit of detection for bacteria was 10^2^ cfu/g of feces. Box-and-whiskers plot indicates high and low values, median and interquartile ranges; each group contained between 8 and 12 mice. Pearson analysis of the bacterial count in faeces (impact of the initial colonization by the wild-type MG1655-s F′ or its derivatives on the capacity of the pathogen (Enteroaggregative *E. coli* 55989a-s) to colonize the mice intestine) and Mann-Whitney analysis of the number of the pathogen CFUs recovered (comparison of pathogen colonization level in mice precolonized with either MG1655-s F′ (control) or its derivatives (*yliE*, *yceP* or *yiaF*)) were performed. Statistically different results (P<0.05), are indicated by an asterisk.

**Figure 5:**
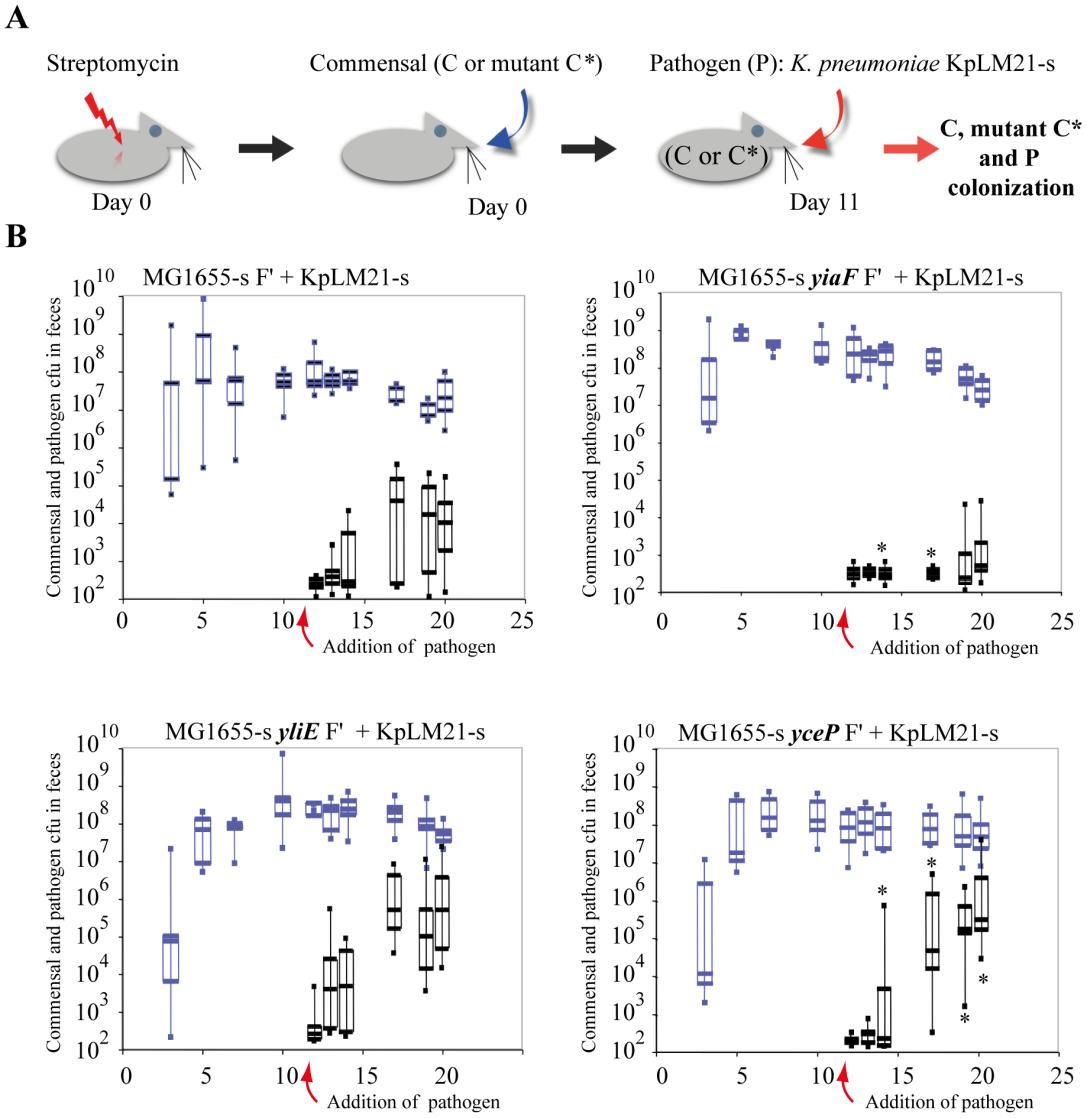
*In vivo* colonization of *E. coli* commensal biofilm by *K. pneumoniae* KpLM21 pathogen. **A** Schematic representation of the experimental procedure. **B** Streptomycin-treated mice were first challenged intragastrically with commensal wild-type MG1655-s F′ (C) or its mutant Δ*yceP*, Δ*yliE*, and Δ*yiaF* derivatives (C*), followed on day 11 by administration of the *K. pneumoniae* KpLM21-s pathogen. The numbers of commensal and pathogen cfus recovered per gram of feces were determined every other day from day 3 to day 20. The lower limit of detection for bacteria was 10^2^ cfu/g of feces. Box-and-whiskers plots indicate high and low values, median and interquartile ranges; each group contained 8 to 12 mice. Pearson analysis of the bacterial count in faeces (impact of the initial colonization by the wild-type MG1655-s F′ or its derivatives on the capacity of the pathogen (*K. pneumoniae* KpLM21-s) to colonize the mice intestine) and Mann-Whitney analysis of the number of the pathogen CFUs recovered (comparison of pathogen colonization level in mice precolonized with either MG1655-s F′ (control) or its derivatives (*yliE*, *yceP* or *yiaF*)) were performed. Statistically different results (P<0.05), are indicated by an asterisk.

To test the *in vivo* role of colonization resistance genes identified via our approach, we used a mouse model of intragastric infection to compare the extent of intestinal colonization by 55989a and KpLM21 pathogens in streptomycin-treated mice, in which enterobacteria such as *E. coli* and *Klebsiella* were shown to colonize the same niches [45], [46]. Streptomycin-treated mice were pre-colonized with wild-type or mutant commensal *E. coli* MG1655 F′ and all three strains efficiently colonized the mouse intestine [47]–[49] (Fig. 4A). We chose to test the role of: i) *yiaF*, which affects *in vitro* colonization resistance to 55989a and was also induced upon KpLM21 colonization (Fig. 1B, and Table 4); ii) *yliE*, which was similarly involved in *in vitro* colonization resistance to 55989a, but was not induced upon KpLM21 colonization (Fig. 1B and Table 4 and Fig. S1); and iii) *yceP*, a gene induced in response to both pathogens, though without detectable *in vitro* effects on 55989a colonization (Fig. 1B and Table 4 and Fig. S1). To perform colonization experiments in streptomycin-treated mice, we first made *E. coli* 55989a and KpLM21 streptomycin-resistant derivatives (respectively, 55989a-s and KpLM21-s). We also introduced *yiaF*, *yliE* and *yceP* mutants into the same streptomycin-resistant derivative of MG1655 F′, (MG1655-s F′) and checked that these strains were not significantly affected in terms of growth and *in vitro* biofilm ability against this background (data not shown). We then determined bacterial counts in feces recovered from individually inoculated mice (n = at least 8 for each strain) and observed that wild-type and MG1655-s F′ *yiaF*, *yliE* and *yceP* mutants reached similar intestinal colonization capacity at day 10 (between 10^7^ to 10^8^ cfu/g of feces) prior to pathogen inoculation (Fig. 4B and 5B). At day 11 post-inoculation, streptomycin-treated mice colonized with wild-type MG1655-s F′ or corresponding *yiaF*, *yliE* and *yceP* mutants were inoculated intragastrically with either 55989a*-s* (Fig. 4) or KpLM21-s (Fig. 5). Determination of Pearson correlation coefficients indicated that there was no (−0.5<P<0.5) or only a moderate (0.5<P<0.8) correlation between colonization levels of wild-type MG1655-sF′ or its mutant derivatives and pathogens (KpLM21-s and 55989a*-s*) at days 10 and 12 post-inoculation by the commensal (wild-type or mutant) strains. Using the non-parametric Mann-Whitney test, comparison from day 12 to day 20 of the numbers of pathogen cfus in the feces of mice previously inoculated with wild-type MG1655-s or *yliE*, *yceP* or *yiaF* mutants indicated that pre-colonization of mice with MG1655-s F′ *yceP*, but not *yliE*, led to statistically significantly increased intestinal colonization by both pathogens (P = 2.3E10^−7^ and P = 0.19, respectively) (Fig. 4B and 5B). In addition, while mice pre-inoculated with MG1655-s F′ *yiaF* displayed lower level (P = 0.01) of KpLM21-s colonization, they showed higher levels (P = 0.01) of *E. coli* 55989a*-s* colonization compared to mice precolonized with wild-type MG1655-s control (Fig. 5B and 4B respectively)

## Discussion

Increased susceptibility to enteric infection after disruption of aerobic gastrointestinal microbiota in *in vivo* models led to the hypothesis that *E. coli* and other facultative aerobes contribute to colonization resistance [9], [10]. In streptomycin-treated mice, a protective effect associated with *E. coli* is partly attributed to production of antibacterial molecules such as colicins and microcins; however, non-producing strains still exhibit protection, suggesting involvement of other bacterial functions in colonization resistance [13]. Here we hypothesized that initial and virtually host-independent competitive interactions between commensal and pathogenic bacteria could be studied in simple *in vitro* experimental settings. We developed a model of commensal *E. coli* biofilms colonized by exogenous pathogens, a situation resembling the proximal intestine environment and the outcome of which partly determines the fate of many gastrointestinal infections.

Transcription profiling of commensal *E. coli* monospecies biofilm with commensal biofilm colonized alone or by the EAEC 55989a pathogen revealed variations in gene expression corresponding to a general response to colonization (self or non-self), potentially corresponding to growth disturbances and substrate competition occurring during introduction of exogenous bacteria into the bacterial community.

Our analysis also revealed variations in gene expression specifically triggered upon colonization by an enteroaggregative *E. coli*. Bacterial capacity to discriminate self from non-self in their environment was shown to result from secretion of quorum sensing molecules, expression of surface autotransporter adhesins enabling bacterial self-recognition and auto-aggregation, and other factors favoring clonality and enabling pathogens to maximize resource utilization and virulence potential [50]–[53]. Our data indicate that discrimination between self and non-self might also be at the origin of colonization resistance, potentially leading to induction of functions controlling bacterial intrusion or reinforcing commensal community cohesion.

We show that colonization of a commensal biofilm by an enteroaggregative *E. coli* induces expression of numerous genes coding for membrane and envelope proteins. One of them is YaeT, also known as BamA, a conserved member of the YaeT/Omp85 family of proteins required for biogenesis of β-barrel outer membrane proteins (OMPs) and involved in contact-dependent inhibition [54]. The strong growth defect displayed by a *yaeT* mutant did not enable us to meaningfully investigate the role of YaeT/BamA. However, *yaeT* expression in commensal bacteria upon pathogen colonization could be due to increased expression of membrane proteins in mixed biofilms. We also observed an intriguingly high proportion of genes located in regions corresponding to defective prophages, including e14 (*stfE*), DLP12 (*ylcE*, *rzpD*), Qin (*hokD*/*relF*, *cspF*) and CP4-6 (*yafX*). Defective prophages are usually considered to be in a state of mutational decay and have lost the ability to sustain a full phage replication cycle [55], [56]. Nevertheless, they often carry functional genes coding, for instance, for cell lysis functions or phage tail-like particles, a special group of bacteriocins composed of fragments of bacteriophages and produced by a number of Enterobacteriaceae and other Gram-negative bacteria [57], [58]. Expression of lytic genes carried by CP4-57- and DLP12-defective phages has recently been associated with biofilm development, suggesting that cell lysis may be an important aspect of *E. coli* biofilm physiology [59]. We show here that deletion of *stfE*, which encodes a putative tail-fiber protein, is involved in colonization resistance, as indicated by the increased colonization of *E. coli* 55989 in biofilm formed by *stfE* mutants. Given that other colonization-induced genes of phage origin are potentially associated with some cell lysis activity (*hokD*, *cspD*, *ylcE*, *rzpD*), this raises the possibility that StfE contributes to excluding incoming *E. coli* 55989a in commensal biofilm. Such a contribution to bacterial weaponry could represent a positive selective force for conservation of a defective prophage gene [60].

A general response to commensal biofilm colonization also involves YceP (BssS) and YliH (BssR), both previously associated with biofilm formation, regulation of indole production and uptake and export of AI-2 through a cAMP-dependent pathway [43], [44]. We observed that, while deletion of *yceP* did not lead to a significant reduction in EAEC pathogen commensal biofilm colonization *in vitro*, it significantly increased *in vivo* colonization of enteroaggregative *E. coli* 55989a-s and *K. pneumoniae* KpLM21-s in mice precolonized with *E. coli* MG1655*ΔyceP* F′. Since *yceP* is induced upon different stresses, including cold, heat shock and oxidative conditions, YceP could contribute to commensal protection in *in vivo* environments [61]–[64]. Finally, colonization of commensal biofilm by EAEC 59989a also leads to overexpression of *yliE*, which is involved in commensal capacity to prevent 55989a pathogen colonization *in vitro*. *yliE* codes for a conserved inner membrane hypothetical 90 kDa protein with an EAL domain associated with phosphodiesterase activity, involved in hydrolysis of the second messenger cyclic di-GMP (c-di-GMP), a key factor in the planktonic-to-biofilm lifestyle switch [36], [65]. Hence, expression of *yliE* in the commensal strain upon pathogen colonization could play a role in c-di-GMP-dependent cell-cell interactions resulting in reduced colonization by incoming pathogens.

Interestingly, while *yliE* and *yiaF* (encoding a conserved inner membrane protein of unknown function) specifically contributed to commensal colonization resistance to the EAEC pathogen *in vitro*, they were also differentially expressed in response to *K. pneumoniae* colonization, along with *yliH* and *yceP.* This suggests the existence of a common genetic response by MG1655 F′ commensal biofilm bacteria to colonization by non-self exogenous bacteria. However, analysis of the *in vivo* contribution of *yliE* and *yiaF* to commensal colonization resistance showed that, while a *yliE* mutation had no influence on EAEC 55989a-s colonization, mice precolonized with a *yiaF* commensal mutant showed increased EAEC 55989a-s colonization. In contrast, we observed decreased *K. pneumoniae* KpLM21-s capacity to be implanted in the intestine of a mouse precolonized with a *ΔyiaF* commensal strain. Hence, although *yliE* and *yiaF* are induced upon colonization of commensal biofilm by the two tested pathogens, they differentially contribute to the *in vivo* colonization phenotype depending on the pathogen. While the exact role of colonization resistance genes identified *in vitro* and *in vivo* currently remains under investigation, it should be noted that strains used in *in vivo* experiments are streptomycin-resistant derivatives of those used for *in vitro* biofilm experiments, thus potentially leading to differences in the colonization phenotype. In addition to genetic background differences, *in vitro* commensal biofilm colonization by pathogens could trigger responses that differ from those of *in vivo* gastrointestinal infection experiments, in which streptomycin-resistant flora might also contribute to regulating pathogen colonization.

In conclusion, the *in vitro*–to-*in vivo* approach described in this study provides a new strategy for studying colonization resistance and unravelling molecular aspects of commensal/pathogen interactions potentially leading to innovative prophylactic intervention in enteric infections.

## Supporting Information

Figure S1 **DNA-array data to** ***in vivo*** **test decision Flow-chart depicting the rational for selection of genes analyzed in the study.**
(DOCX)

Figure S2 **Estimate of biofilm biomass before inoculation with pathogen.** Microfermentors were inoculated with commensal strain MG1655 F′ (C) or with indicated devivative mutants. After 6 h of growth, biofilm that developed on the glass slide was resuspended in 10 ml of minimal media and recovered bacterial count was estimated by serial dilution and cfu count. Results are average of at least 6 replicates ± standard deviation of the mean. Star indicates a mutant for which initial biofilm formation significantly differed from that of the wild type, P≤0.05.
(DOCX)

Table S1 Genes over-expressed or repressed in response to colonization of MG1655 F′ biofilm by pathogenic 55989*a*.
(DOCX)

Table S2 Genes induced upon self-colonization (C+C) of commensal biofilm.
(DOCX)

Table S3 Genes repressed upon self-colonization (C+C) of commensal biofilm.
(DOCX)

Table S4 Genes induced upon colonization by exogenous pathogen (C+P).
(DOCX)

Table S5 Genes repressed upon colonization by exogenous pathogen (C+P).
(DOCX)

Table S6 Primers used in this study.
(DOCX)
