# Supplementary material for: Identification of Commensal Escherichia coli Genes Involved in Biofilm Resistance to Pathogen Colonization
Source: PLoS One. 2013 May 7;8(5):e61628. doi: 10.1371/journal.pone.0061628 (PMC3646849; doi:10.1371/journal.pone.0061628)

**Figure S1**: **DNA-array data to in vivo test decision Flow-chart depicting the rational for selection of genes analyzed in the study.**


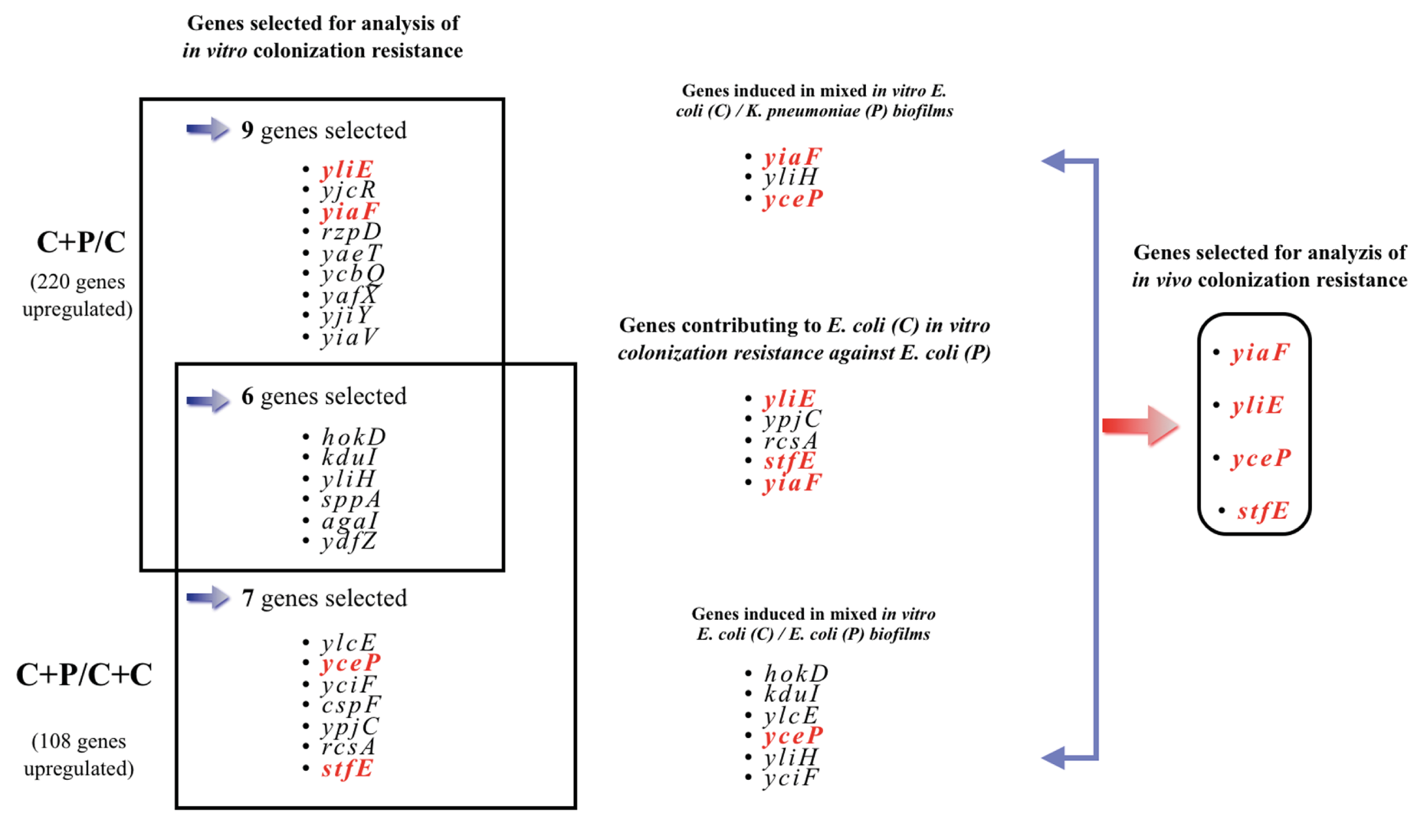

Supplement: Figure S1 — DNA-array data to in vivo test decision Flow-chart depicting the rational for selection of genes analyzed in the study. (DOCX) [file pone.0061628.s001.docx]
