# Supplementary material for: Identification of Commensal Escherichia coli Genes Involved in Biofilm Resistance to Pathogen Colonization
Source: PLoS One. 2013 May 7;8(5):e61628. doi: 10.1371/journal.pone.0061628 (PMC3646849; doi:10.1371/journal.pone.0061628)

**Figure S2**: **Estimate of biofilm biomass before inoculation with pathogen.** Microfermentors were inoculated with commensal strain MG1655 F’ (C) or with indicated devivative mutants. After 6 h of growth, biofilm that developed on the glass slide was resuspended in 10 ml of minimal media and recovered bacterial count was estimated by serial dilution and cfu count. Results are average of at least 6 replicates ± standard deviation of the mean. Star indicates a mutant for which initial biofilm formation significantly differed from that of the wild type, P≤0.05.


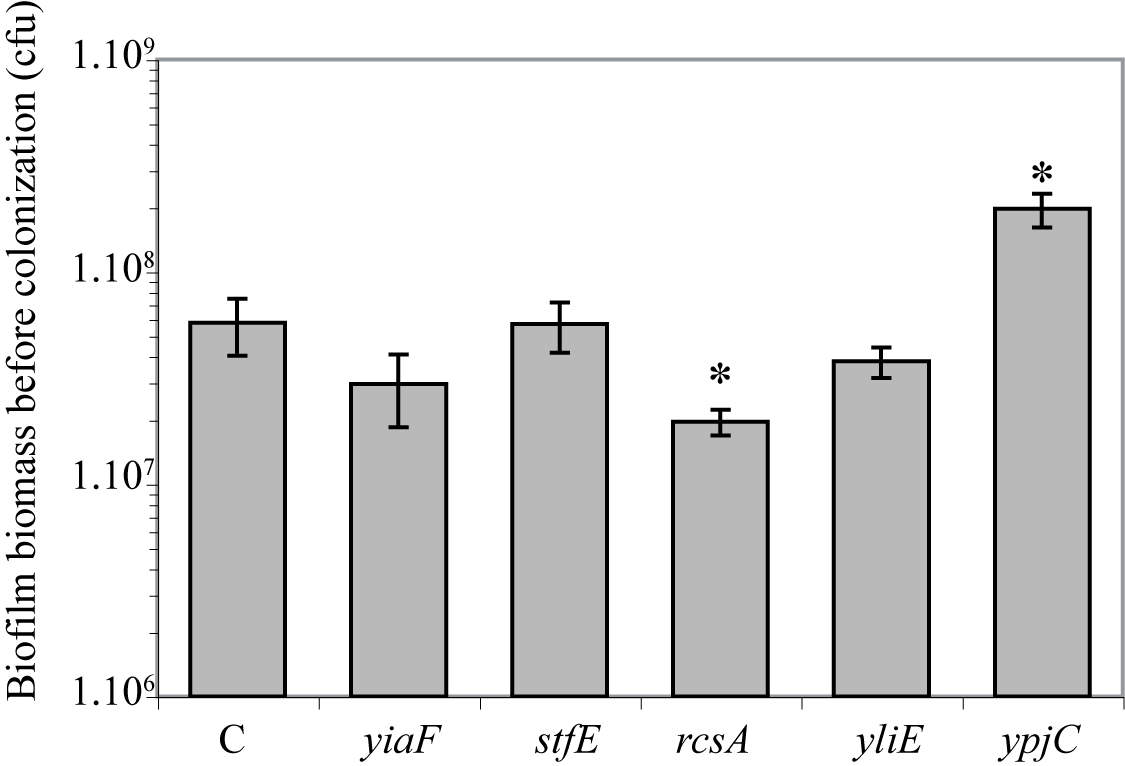

Supplement: Figure S2 — Estimate of biofilm biomass before inoculation with pathogen. Microfermentors were inoculated with commensal strain MG1655 F′ (C) or with indicated devivative mutants. After 6 h of growth, biofilm that developed on the glass slide was resuspended in 10 ml of minimal media and recovered bacterial count was estimated by serial dilution and cfu count. Results are average of at least 6 replicates ± standard deviation of the mean. Star indicates a mutant for which initial biofilm formation significantly differed from that of the wild type, P≤0.05. (DOCX) [file pone.0061628.s002.docx]
