# Supplementary material for: Identification of Commensal Escherichia coli Genes Involved in Biofilm Resistance to Pathogen Colonization
Source: PLoS One. 2013 May 7;8(5):e61628. doi: 10.1371/journal.pone.0061628 (PMC3646849; doi:10.1371/journal.pone.0061628)
Supplement: Table S1 — Genes over-expressed or repressed in response to colonization of MG1655 F′ biofilm by pathogenic 55989a. (DOCX) [file pone.0061628.s003.docx]

**Table S1**: **Genes over-expressed or repressed in response to colonization of MG1655 F’ biofilm by pathogenic 55989*a*.**

| **Overexpressed genes** | | | | | | | | |  | | Repressed genes | | | | | | | | | | | | | |  |
| --- | --- | --- | --- | --- | --- | --- | --- | --- | --- | --- | --- | --- | --- | --- | --- | --- | --- | --- | --- | --- | --- | --- | --- | --- | --- |
| **Gene name** | | *C+P / C^c^* | | | | *C+P / C+C^d^* | | **Function-description^f^** |  | | Gene Name | | | | *C+P / C^c^* | | | | *C+P / C+C^d^* | | | | **Function-description^f^** | |  |
| **a** | **b** | | **Rank^e^** | | **ratio** | **Rank^e^** | **ratio** |  |  | | **a** | | **b** | | **Rank^e^** | | **ratio** | | **Rank^e^** | | **ratio** | |  | |  |
| Information storage and processing | | | | | | | | |  | | Information storage and processing | | | | | | | | | | | | | |  |
| J: Translation, ribosomal structure and biogenesis | | | | | | | | |  | | J: Translation, ribosomal structure and biogenesis | | | | | | | | | | | | | |  |
| *lysU* | b4129 | | 12 | 1.74 | | 89 | 1.39 | lysine tRNA synthetase |  | | *rplK* | | b3983 | | 58 | | 0.70 | | 28 | | 0.74 | | 50S ribosomal subunit protein L11 | |  |
| *yabO* | b0058 | | 206 | 1.22 | | 94 | 1.32 | pseudouridylate synthase |  | |  | |  | |  | |  | |  | |  | |  | |  |
|  | | | | | | | | |  | | K: Transcription | | | | | | | | | | | | | |  |
|  |  | |  |  | |  |  |  |  | | *yhhY* | | b3441 | | 1 | | 0.30 | | 15 | | 0.66 | | predicted acetyltransferase | |  |
|  | | | | | | | | |  | | L: DNA replication, recombination and repair | | | | | | | | | | | | | |  |
|  |  | |  |  | |  |  |  |  | | *b4272* | | b4272 | | 76 | | 0.73 | | 48 | | 0.81 | | IS2 insertion element repressor InsA | |  |
|  |  | |  |  | |  |  |  |  | | *rep* | | b3778 | | 103 | | 0.80 | | 52 | | 0.82 | | rep helicase | |  |
| Cellular processes | | | | | | | | |  | | Cellular processes | | | | | | | | | | | | | |  |
| O: Posttranslational modification, protein turnover, chaperones | | | | | | | | |  | | O: Posttranslational modification, protein turnover, chaperones | | | | | | | | | | | | | |  |
| *sppA* | b1766 | | 6 | 1.91 | | 38 | 1.92 | protease IV |  | | *hypF* | | b2712 | | 18 | | 0.61 | | 41 | | 0.78 | | hydrogenase maturation protein, carbamoyltransferase | |  |
| N: Cell motility and secretion | | | | | | | | |  | |  | |  | |  | |  | |  | |  | |  | |  |
| *sfmH* | b0533 | | 47 | 1.49 | | 16 | 2.18 | involved in fimbrial assembly |  | |  | |  | |  | |  | |  | |  | |  | |  |
| T: Signal transduction mechanisms | | | | | | | | |  | |  | |  | |  | |  | |  | |  | |  | |  |
| *fnr* | b1334 | | 84 | 1.40 | | 93 | 1.35 | Fnr |  | |  | |  | |  | |  | |  | |  | |  | |  |
| *ycdT* | b1025 | | 50 | 1.48 | | 55 | 1.72 | diguanylate cyclase, regulates motility |  | |  | |  | |  | |  | |  | |  | |  | |  |
| U: Intracellular trafficking, secretion and vesicular transport | | | | | | | | |  | | U: Intracellular trafficking, secretion and vesicular transport | | | | | | | | | | | | | |  |
| *secE* | b3981 | | 158 | 1.30 | | 95 | 1.32 | preprotein translocase |  | | *yjaI* | | b4002 | | 69 | | 0.71 | | 35 | | 0.76 | | hypothetical protein | |  |
| Metabolism | | | | | | | | |  | | Metabolism | | | | | | | | | | | | | |  |
| C: Energy production and conversion | | | | | | | | |  | | C: Energy production and conversion | | | | | | | | | | | | | |  |
| *ydhY* | b1674 | | 81 | 1.41 | | 102 | 1.27 | putative oxidoreductase Fe-S subunit |  | | *hyaD* | | b0975 | | 68 | | 0.71 | | 49 | | 0.81 | | HyaD | |  |
|  |  | |  |  | |  |  |  |  | | *yibA* | | b3594 | | 74 | | 0.73 | | 34 | | 0.76 | | predicted lyase containing HEAT-repaet | |  |
| G: Carbohydrate transport and metabolism | | | | | | | | |  | | G: Carbohydrate transport and metabolism | | | | | | | | | | | | | |  |
| *agaI* | b3141 | | 7 | 1.89 | | 9 | 2.37 | putative galactosamine-6-phosphate isomerase |  | | *gatY* | | b2096 | | 84 | | 0.75 | | 38 | | 0.77 | | tagatose-bisphosphate aldolase 1 | |  |
| *agaY* | b3137 | | 70 | 1.43 | | 59 | 1.67 | tagatose-bisphosphate aldolase 2 |  | | *ybjJ* | | b0845 | | 61 | | 0.70 | | 46 | | 0.80 | | putative DEOR-type transcriptional regulator | |  |
| *araF* | b1901 | | 32 | 1.55 | | 83 | 1.43 | L-arabinose-binding periplasmic protein |  | |  | |  | |  | |  | |  | |  | |  | |  |
| *frvX* | b3898 | | 220 | 1.16 | | 107 | 1.18 | frv operon protein |  | |  | |  | |  | |  | |  | |  | |  | |  |
| *kduI* | b2843 | | 2 | 2.88 | | 3 | 2.57 | homolog of pectin-degrading enzyme 5-keto 4-deoxyuronate isomerase |  | |  | |  | |  | |  | |  | |  | |  | |  |
| E: Amino acid transport and metabolism | | | | | | | | | |  | | E: Amino acid transport and metabolism | | | | | | | | | | | | | |
| *aroD* | b1693 | | 10 | 1.75 | | 52 | 1.74 | 3-dehydroquinate dehydratase | |  | | *gltL* | | b0652 | | 86 | | 0.75 | | 25 | | 0.73 | | GltL | |
| *carA* | b0032 | | 211 | 1.21 | | 105 | 1.20 | carbamoyl-phosphate synthetase | |  | |  | |  | |  | |  | |  | |  | |  | |
| *dsdA* | b2366 | | 8 | 1.81 | | 35 | 1.98 | D-serine dehydratase | |  | |  | |  | |  | |  | |  | |  | |  | |
|  | | | | | | | | | |  | | F: Nucleotide transport and metabolism | | | | | | | | | | | | | |
|  |  | |  |  | |  |  |  | |  | | *ushA* | | b0480 | | 27 | | 0.62 | | 21 | | 0.68 | | UDP-sugar hydrolase (5'-nucleotidase) | |
| H: Coenzyme metabolism | | | | | | | | | |  | | H: Coenzyme metabolism | | | | | | | | | | | | | |
|  |  | |  |  | |  |  |  | |  | | *csdL* | | b2812 | | 30 | | 0.63 | | 16 | | 0.66 | | CSD sulfur transfer protein | |
| Poorly characterized | | | | | | | | | |  | | Poorly characterized | | | | | | | | | | | | | |
| R: General function prediction only | | | | | | | | | |  | | R: General function prediction only | | | | | | | | | | | | | |
| *stpA* | b2669 | | 168 | 1.28 | | 86 | 1.42 | StpA | |  | | *ychK* | | b1234 | | 65 | | 0.71 | | 23 | | 0.71 | | hypothetical protein | |
| *yegI* | b2070 | | 71 | 1.43 | | 97 | 1.29 | putative chaperonin | |  | | *viaA* | | b3745 | | 80 | | 0.74 | | 59 | | 0.85 | | stimulator of RavA ATPase activity | |
|  |  | |  |  | |  |  |  | |  | | *yjgR* | | b4263 | | 17 | | 0.60 | | 30 | | 0.75 | | predicted ATPase | |
|  |  | |  |  | |  |  |  | |  | | *yoaE* | | b1816 | | 13 | | 0.59 | | 9 | | 0.63 | | putative transport protein | |
|  | | | | | | | | | |  | | S: Function unknown | | | | | | | | | | | | | |
|  |  | |  |  | |  |  |  | |  | | *yaeQ* | | b0190 | | 31 | | 0.63 | | 26 | | 0.73 | | hypothetical protein | |
|  |  | |  |  | |  |  |  | |  | | *ygjQ* | | b3086 | | 50 | | 0.68 | | 19 | | 0.67 | | hypothetical protein | |
|  |  | |  |  | |  |  |  | |  | | *yhbP* | | b3154 | | 90 | | 0.76 | | 12 | | 0.65 | | hypothetical protein | |
|  |  | |  |  | |  |  |  | |  | | *yqjE* | | b3099 | | 75 | | 0.73 | | 47 | | 0.80 | | conserved protein inner membrane | |
| No COG classification | | | | | | | | | |  | | No COG classification | | | | | | | | | | | | | |
| *relF* | b1562 | | 1 | 4.85 | | 1 | 7.52 | RelF | |  | | *ygdB* | | b2824 | | 11 | | 0.58 | | 2 | | 0.54 | | hypothetical protein | |
| *ydfZ* | b1541 | | 9 | 1.79 | | 2 | 2.77 | hypothetical protein | |  | | *ygjV* | | b3090 | | 33 | | 0.64 | | 3 | | 0.55 | | inner membrane protein | |
| *yliH* | b0836 | | 3 | 2.47 | | 18 | 2.15 | regulator of biofilm formation | |  | | *yibQ* | | b3614 | | 57 | | 0.70 | | 17 | | 0.66 | | putative nucleoside (IDP) diphosphatase | |
| *smf_2 (f253)* | b3286 | | 45 | 1.49 | | 100 | 1.28 |  | |  | | *yjaH* | | b4001 | | 12 | | 0.58 | | 10 | | 0.64 | | hypothetical protein | |
|  |  | |  |  | |  |  |  | |  | | *yjis* | | b4341 | | 16 | | 0.60 | | 13 | | 0.65 | | hypothetical protein | |

**a.** Gene names according to *E.coli EcoCyc* database (www.ecocyc.org)

**b.** Gene names according to Blattner nomenclature (http://www.genome.wisc.edu/sequencing/k12.htm#gen).

**c.** Comparison of gene expression in MG1655 F’ + 55989*a* biofilm (C + P) versus gene expression in MG1655 F’ biofilm (C) expressed as ratio.

**d.** Comparison of gene expression in MG1655 F’ + 55989*a* biofilm (C + P) versus gene expression in MG1655 F’ + MG1655 F’ biofilm (C + C) expressed as ratio.

**e.** Rank position; 1 = the most induced or repressed genes in the mixed biofilm (C+P) versus the non-infected commensal (C) or the self-infected (C + C) biofilm.

**f.** Function description according to COG functional categories annotation system used by the NCBI (http://www.ncbi.nlm.nih.gov/COG).
