# Supplementary material for: Identification of Commensal Escherichia coli Genes Involved in Biofilm Resistance to Pathogen Colonization
Source: PLoS One. 2013 May 7;8(5):e61628. doi: 10.1371/journal.pone.0061628 (PMC3646849; doi:10.1371/journal.pone.0061628)
Supplement: Table S2 — Genes induced upon self-colonization (C+C) of commensal biofilm. (DOCX) [file pone.0061628.s004.docx]

**Table S2**: **Genes induced upon self-colonization (C+C) of commensal biofilm.**

| **Gene name** | | **Rank^c^** | *C + C* **/ C^d^** | **Function-description^e^** |
| --- | --- | --- | --- | --- |
| **a** | **b** |  |  |  |
| Information storage and processing | | | | |
| J: Translation, ribosomal structure and biogenesis | | | | |
| def | b3287 | 29 | 1.51 | peptide deformylase |
| fusA | b3340 | 27 | 1.52 | GTP-binding protein chain elongation factor EF-G |
| rimL | b1427 | 141 | 1.24 | acetyl transferase |
| yfiF | b2581 | 161 | 1.14 | hypothetical protein |
| K: Transcription | | | | |
| cbl | b1987 | 14 | 1.59 | Cbl |
| glcC | b2980 | 101 | 1.33 | GlcC |
| hepA | b0059 | 12 | 1.60 | putative ATP-dependent RNA helicase |
| rho | b3783 | 83 | 1.38 | transcription termination factor Rho |
| rpoB | b3987 | 121 | 1.29 | RNA polymerase beta subunit |
| rpoN | b3202 | 146 | 1.23 | RpoN |
| yagI | b0272 | 11 | 1.63 | putative regulator |
| yahB | b0316 | 51 | 1.43 | putative transcriptional regulator LYSR-type |
| ybcM | b0546 | 119 | 1.29 | putative ARAC-type regulatory protein |
| ycjW | b1320 | 94 | 1.35 | putative LACI-type transcriptional regulator |
| ydcR | b1439 | 36 | 1.48 | hypothetical protein |
| yehV | b2127 | 152 | 1.21 | putative transcriptional regulator |
| ygbI | b2735 | 109 | 1.31 | putative DEOR-type transcriptional regulator |
| yjcT | b4084 | 75 | 1.39 | putative NAGC-like transcriptional regulator |
| ynfL | b1595 | 54 | 1.43 | putative transcriptional regulator LYSR-type |
| L: DNA replication, recombination and repair | | | | |
| dnaB | b4052 | 44 | 1.46 | replicative DNA helicase |
| insA_2 | b0265 | 1 | 2.49 | InsA |
| priA | b3935 | 10 | 1.68 | primosomal protein N' |
| priB | b4201 | 105 | 1.32 | primosomal replication protein N |
| Cellular processes | | | | |
| D: Cell division and chromosome partitioning | | | | |
| ftsL | b0083 | 15 | 1.59 | cell division protein |
| mreB | b3251 | 124 | 1.29 | penicillin binding protein 3 |
| O: Posttranslational modification, protein turnover, chaperones | | | | |
| hhoA | b3234 | 77 | 1.38 | serine endoprotease |
| hscA | b2526 | 38 | 1.47 | HscA |
| htpG | b0473 | 87 | 1.37 | chaperone Hsp90 |
| hypD | b2729 | 151 | 1.21 | HypD |
| yegQ | b2081 | 145 | 1.24 | hypothetical protein |
| yncG | b1454 | 134 | 1.27 | putative transferase |
| M: Cell envelope biogenesis, outer membrane | | | | |
| acrE | b3265 | 35 | 1.48 | AcrE |
| aefA | b0465 | 90 | 1.36 | putative alpha helix protein |
| dniR | b0211 | 59 | 1.42 | cytochrome c552 |
| lepA | b2569 | 95 | 1.35 | GTP-binding elongation factor |
| mepA | b2328 | 98 | 1.34 | murein DD-endopeptidase |
| pbpC | b2519 | 42 | 1.46 | putative peptidoglycan enzyme |
| ydeU | b1509 | 120 | 1.29 | putative ATP-binding component of a transport system and adhesin protein |
| yrbM | b3208 | 32 | 1.50 | putative peptidoglycan enzyme |
| N: Cell motility and secretion | | | | |
| hofH | b3329 | 55 | 1.43 | putative general protein secretion protein |
| ycbR | b0939 | 70 | 1.40 | putative chaperone |
| yraI | b3143 | 140 | 1.25 | putative chaperone |
| P: Inorganic ion transport and metabolism | | | | |
| aslA | b3801 | 114 | 1.30 | arylsulfatase |
| kup | b3747 | 156 | 1.19 | low affinity potassium transport system |
| narU | b1469 | 76 | 1.38 | nitrite extrusion protein 2 |
| phnM | b4095 | 69 | 1.40 | PhnM |
| phoA | b0383 | 47 | 1.43 | alkaline phosphatase |
| pitA | b3493 | 71 | 1.39 | low-affinity phosphate transport |
| tauA | b0365 | 133 | 1.27 | TauA |
| tauC | b0367 | 74 | 1.39 | TauC |
| trkG | b1363 | 62 | 1.41 | trk system potassium uptake |
| T: Signal transduction mechanisms | | | | |
| creC | b4399 | 131 | 1.27 | CreC |
| phoH | b1020 | 45 | 1.45 | PhoH |
| phoQ | b1129 | 16 | 1.59 | PhoQ |
| phoR | b0400 | 148 | 1.23 | positive and negative sensor protein for pho regulon |
| rstB | b1609 | 66 | 1.40 | sensor histidine protein kinase |
| ypdA | b2380 | 82 | 1.38 | putative sensor protein |
| V: Defense mechanisms | | | | |
| sapF | b1290 | 116 | 1.30 | putative ATP-binding protein of peptide transport system |
| Metabolism | | | | |
| C: Energy production and conversion | | | | |
| acnB | b0118 | 84 | 1.38 | aconitate hydrase B |
| adhC | b0356 | 115 | 1.30 | alcohol dehydrogenase class III |
| aldA | b1415 | 13 | 1.60 | aldehyde dehydrogenase, NAD-linked |
| cydC | b0886 | 78 | 1.38 | ATP-binding component of cytochrome-related transport |
| cydD | b0887 | 33 | 1.49 | ATP-binding component of cytochrome-related transport |
| dmsB | b0895 | 24 | 1.54 | anaerobic dimethyl sulfoxide reductase subunit B |
| eutE | b2455 | 9 | 1.74 | EutE |
| fdnG | b1474 | 107 | 1.32 | formate dehydrogenase-N, nitrate-inducible, alpha subunit |
| fdrA | b0518 | 89 | 1.37 | FdrA |
| gor | b3500 | 61 | 1.42 | glutathione oxidoreductase |
| hybO | b2997 | 126 | 1.28 | putative hydrogenase subunit |
| sfcA | b1479 | 85 | 1.38 | NAD-linked malate dehydrogenase |
| sucB | b0727 | 144 | 1.24 | 2-oxoglutarate dehydrogenase |
| ynfE | b1587 | 135 | 1.27 | putative oxidoreductase major subunit |
| G: Carbohydrate transport and metabolism | | | | |
| celF | b1734 | 50 | 1.43 | phospho-beta-glucosidase |
| dsdX | b2365 | 73 | 1.39 | transport system permease |
| frvR | b3897 | 132 | 1.27 | putative frv operon regulatory protein |
| gntV | b4268 | 142 | 1.24 | gluconate kinase |
| gsk | b0477 | 136 | 1.26 | inosine-guanosine kinase |
| hrsA | b0731 | 26 | 1.52 | HrsA |
| melA | b4119 | 3 | 1.94 | alpha-galactosidase |
| melB | b4120 | 41 | 1.47 | melibiose permease II |
| mtlD | b3600 | 122 | 1.29 | mannitol-1-phosphate dehydrogenase |
| otsA | b1896 | 19 | 1.57 | trehalose-6-phosphate synthase |
| ppsA | b1702 | 8 | 1.78 | phosphoenolpyruvate synthase |
| ptsA | b3947 | 118 | 1.30 | PEP-protein phosphotransferase system enzyme I |
| rhaA | b3903 | 159 | 1.17 | L-rhamnose isomerase |
| xylF | b3566 | 72 | 1.39 | xylose binding protein transport system |
| ydfI | b1542 | 96 | 1.35 | putative oxidoreductase |
| yeiQ | b2172 | 28 | 1.52 | putative oxidoreductase |
| ygbN | b2740 | 158 | 1.18 | putative transport protein |
| yjhF | b4296 | 37 | 1.47 | putative transport system permease |
| ypdD | b2383 | 7 | 1.81 | putative PTS system enzyme IIA component, enzyme I |
| E: Amino acid transport and metabolism | | | | |
| argE | b3957 | 155 | 1.20 | acetylornithine deacetylase |
| betA | b0311 | 18 | 1.57 | choline dehydrogenase |
| cadB | b4132 | 57 | 1.43 | transport of lysine/cadaverine |
| gatD | b2091 | 99 | 1.34 | galactitol-1-phosphate dehydrogenase |
| gltD | b3213 | 157 | 1.18 | glutamate synthase small subunit |
| goaG | b1302 | 39 | 1.47 | 4-aminobutyrate aminotransferase |
| ilvC | b3774 | 81 | 1.38 | ketol-acid reductoisomerase |
| metB | b3939 | 130 | 1.27 | cystathionine gamma-synthase |
| poxB | b0871 | 111 | 1.31 | pyruvate oxidase |
| ptrB | b1845 | 21 | 1.56 | protease II |
| putA | b1014 | 149 | 1.23 | proline dehydrogenase |
| sdaB | b2797 | 147 | 1.23 | L-serine dehydratase |
| ybiK | b0828 | 123 | 1.29 | putative asparaginase |
| ycaM | b0899 | 31 | 1.51 | putative transport |
| yddR | b1486 | 79 | 1.38 | putative transport system permease protein |
| ydjJ | b1774 | 112 | 1.31 | putative oxidoreductase |
| yehY | b2130 | 127 | 1.28 | putative transport system permease protein |
| yeiT | b2146 | 106 | 1.32 | putative oxidoreductase |
| yhdY | b3270 | 139 | 1.25 | putative transport system permease protein |
| F: Nucleotide transport and metabolism | | | | |
| nrdB | b2235 | 80 | 1.38 | ribonucleoside-diphosphate reductase 1, beta subunit, B2 |
| nupC | b2393 | 103 | 1.33 | NupC |
| purB | b1131 | 43 | 1.46 | adenylosuccinate lyase |
| pyrC | b1062 | 138 | 1.26 | dihydro-orotase |
| xapA | b2407 | 68 | 1.40 | xanthosine phosphorylase |
| H: Coenzyme metabolism | | | | |
| btuB | b3966 | 143 | 1.24 | BtuB |
| menC | b2261 | 104 | 1.33 | o-succinylbenzoyl-CoA synthase |
| ribB | b3041 | 91 | 1.36 | 3,4 dihydroxy-2-butanone-4-phosphate synthase |
| ydaH | b1336 | 40 | 1.47 | putative pump protein |
| I: Lipid metabolism | | | | |
| accA | b0185 | 65 | 1.40 | acetyl-CoA carboxylase |
| caiA | b0039 | 88 | 1.37 | putative carnitine operon oxidoreductase |
| prpE | b0335 | 108 | 1.31 | putative propionyl-CoA synthetase |
| yafH | b0221 | 154 | 1.20 | putative acyl-CoA dehydrogenase |
| ybbQ | b0509 | 93 | 1.35 | putative oxidoreductase |
| yqeF | b2844 | 34 | 1.48 | putative acyltransferase |
| Q: Secondary metabolites biosynthesis. transport and catabolism | | | | |
| yrbE | b3194 | 125 | 1.29 | hypothetical protein |
| Poorly characterized | | | | |
| R: General function prediction only | | | | |
| hipA | b1507 | 49 | 1.43 | HipA |
| ybgL | b0713 | 5 | 1.86 | putative lactam utilization protein |
| yeiR | b2173 | 100 | 1.33 | hypothetical protein |
| yidK | b3679 | 129 | 1.28 | putative cotransporter |
| yieF | b3713 | 117 | 1.30 | hypothetical protein |
| yjdA | b4109 | 4 | 1.92 | putative vimentin |
| ypfI | b2474 | 17 | 1.58 | hypothetical protein |
| ytfL | b4218 | 63 | 1.41 | putative transport protein |
| S: Function unknown | | | | |
| wcaK | b2045 | 48 | 1.43 | putative galactokinase |
| yhgL | b3421 | 92 | 1.35 | hypothetical protein |
| yifE | b3764 | 25 | 1.53 | hypothetical protein |
| yjbI | b4038 | 86 | 1.37 | hypothetical protein |
| yjiN | b4336 | 137 | 1.26 | hypothetical protein |
| No COG classification | | | | |
| abgA | b1338 | 128 | 1.28 | putative aminohydrolase |
| arsB | b3502 | 150 | 1.21 | arsenical pump membrane protein |
| eutB | b2441 | 153 | 1.21 | ethanolamine ammonia-lyase, heavy chain |
| gapC_2 | b1416 | 110 | 1.31 |  |
| insA_7 | b4294 | 2 | 2.43 | IS1 protein InsA |
| kdgT | b3909 | 102 | 1.33 | 2-keto-3-deoxy-D-gluconate transport system |
| nanT | b3224 | 23 | 1.54 | sialic acid transporter |
| pspD | b1307 | 52 | 1.43 | phage shock protein |
| pssA | b2585 | 56 | 1.43 | phosphatidylserine synthase |
| thiG | b3991 | 113 | 1.30 | ThiG |
| ybbD | b0500 | 6 | 1.84 | hypothetical protein |
| ybeL | b0643 | 60 | 1.42 | putative alpha helical protein |
| ybfM | b0681 | 22 | 1.56 | hypothetical protein |
| ycjM | b1309 | 58 | 1.43 | putative polysaccharide hydrolase |
| yfdI | b2352 | 30 | 1.51 | putative ligase |
| yghJ | b2974 | 20 | 1.57 | putative endoglucanase |
| yhdW | b3268 | 97 | 1.34 |  |
| yhiM | b3491 | 64 | 1.41 | hypothetical protein |
| yhjU | b3538 | 53 | 1.43 | hypothetical protein |
| yidE | b3685 | 160 | 1.16 | putative transport protein |
| yjiT | b4342 | 67 | 1.40 | hypothetical protein |
| ynaA | b1368 | 46 | 1.45 | putative alpha helix protein |

**a.** Gene names according to *E. coli* Colibri database (http://genolist.pasteur.fr/Colibri/).

**b.** Gene names according to Blattner nomenclature (http://www.genome.wisc.edu/sequencing/k12.htm#gen).

**c.** Rank position; 1 = most overexpressed gene in self-infected biofilm (C+C) versus non-infected monospecies commensal (C) biofilm.

**d.** Ratio of gene expression in *E. coli* MG1655 F’ + MG1655 F’ biofilm (C + C) versus gene expression in MG1655 F’ biofilm (C).

**e.** Function description according to COG functional categories annotation system used by the NCBI (http://www.ncbi.nlm.nih.gov/COG).
