# Supplementary material for: Identification of Commensal Escherichia coli Genes Involved in Biofilm Resistance to Pathogen Colonization
Source: PLoS One. 2013 May 7;8(5):e61628. doi: 10.1371/journal.pone.0061628 (PMC3646849; doi:10.1371/journal.pone.0061628)
Supplement: Table S3 — Genes repressed upon self-colonization (C+C) of commensal biofilm. (DOCX) [file pone.0061628.s005.docx]

**Table S3**: **Genes repressed upon self-colonization (C+C) of commensal biofilm.**

| Gene name | | **Rank^c^** | *C + C* **/ C^d^** | Function-description^e^ |
| --- | --- | --- | --- | --- |
| a | b |  |  |  |
| Information storage and processing | | | | |
| J: Translation. ribosomal structure and biogenesis | | | | |
| rplS | b2606 | 168 | 0.78 | 50S ribosomal subunit protein L19 |
| rplY | b2185 | 84 | 0.56 | 50S ribosomal subunit protein L25 |
| rpmG | b3636 | 160 | 0.75 | 50S ribosomal subunit protein L33 |
| rpsP | b2609 | 181 | 0.84 | 30S ribosomal subunit protein S16 |
| tsf | b0170 | 79 | 0.55 | protein chain elongation factor EF-Ts |
| yaeJ | b0191 | 178 | 0.83 | hypothetical protein |
| ybjF | b0859 | 131 | 0.66 | hypothetical protein |
| yhbY | b3180 | 98 | 0.60 | hypothetical protein |
| K: Transcription | | | | |
| abgR | b1339 | 146 | 0.70 | putative transcriptional regulator LYSR-type |
| dicA | b1570 | 18 | 0.42 | DicA |
| envY | b0566 | 51 | 0.49 | envelope protein |
| nusB | b0416 | 37 | 0.47 | transcription termination |
| rpoA | b3295 | 16 | 0.41 | RNA polymerase alpha subunit |
| umuD | b1183 | 129 | 0.66 | UmuD |
| yeaM | b1790 | 170 | 0.79 | putative ARAC-type regulatory protein |
| yfaX | b2248 | 32 | 0.45 | putative regulator |
| yfjR | b2634 | 143 | 0.69 | hypothetical protein |
| ygeH | b2852 | 68 | 0.52 | putative invasion protein |
| yggD | b2929 | 8 | 0.38 | putative transcriptional regulator |
| yjeB | b4178 | 10 | 0.40 | hypothetical protein |
| yjgS | b4264 | 97 | 0.60 | L-idonate transcriptional regulator |
| L: DNA replication. recombination and repair | | | | |
| helD | b0962 | 183 | 0.87 | DNA helicase IV |
| recD | b2819 | 14 | 0.40 | RecD |
| recT | b1349 | 15 | 0.40 | recombinase |
| topB | b1763 | 155 | 0.74 | DNA topoisomerase III |
| yfjY | b2644 | 87 | 0.57 | putative DNA repair protein |
| Cellular processes | | | | |
| D: Cell division and chromosome partitioning | | | | |
| ygbQ | b2748 | 138 | 0.68 | hypothetical protein |
| O: Posttranslational modification. protein turnover. chaperones | | | | |
| msrA | b4219 | 108 | 0.61 | peptide methionine sulfoxide reductase |
| yaaD | b0028 | 171 | 0.79 | putative FKBX-type 16KD peptidyl-prolyl cis-trans isomerase |
| M: Cell envelope biogenesis. outer membrane | | | | |
| mraY | b0087 | 102 | 0.60 | phospho-N-acetylmuramoyl-pentapeptide transferase |
| nlpC | b1708 | 127 | 0.66 | lipoprotein |
| N: Cell motility and secretion | | | | |
| ydeR | b1503 | 34 | 0.46 | putative fimbrial-like protein |
| P: Inorganic ion transport and metabolism | | | | |
| cysP | b2425 | 159 | 0.74 | thiosulfate binding protein |
| modB | b0764 | 92 | 0.59 | molybdate transport permease protein |
| phnE | b4103 | 3 | 0.33 | hypothetical protein |
| ycdN | b1016 | 85 | 0.56 | high-affinity iron permease |
| yheL | b3343 | 43 | 0.48 | hypothetical protein |
| znuC | b1858 | 100 | 0.60 | putative ATP-binding component of a transport system |
| T: Signal transduction mechanisms | | | | |
| cheY | b1882 | 61 | 0.51 | CheY |
| chpB | b4225 | 151 | 0.72 | ChpB |
| chpR | b2783 | 21 | 0.43 | ChpR |
| evgA | b2369 | 162 | 0.76 | EvgA |
| sixA | b2340 | 140 | 0.68 | hypothetical protein |
| ygiX | b3025 | 82 | 0.56 | quorum sensing regulator B |
| yhjH | b3525 | 153 | 0.72 | hypothetical protein |
| ylaB | b0457 | 156 | 0.74 | hypothetical protein |
| U: Intracellular trafficking, secretion and vesicular transport | | | | |
| ccmD | b2198 | 77 | 0.54 | heme exporter protein C |
| yheI | b3331 | 101 | 0.60 | putative export protein I |
| V: Defense mechanisms | | | | |
| yibH | b3597 | 154 | 0.73 | putative membrane protein |
| Metabolism | | | | |
| C: Energy production and conversion | | | | |
| yccM | b0992 | 95 | 0.59 | hypothetical protein |
| yhaA | b3115 | 167 | 0.78 | putative kinase |
| G: Carbohydrate transport and metabolism | | | | |
| agaD | b3140 | 83 | 0.56 | PTS system, N-acetylglucosamine enzyme IID component 1 |
| agaW | b3134 | 175 | 0.81 | PTS system N-acetylgalactosameine-specific IIC component 2 |
| celA | b1738 | 111 | 0.62 | CelA |
| cmtA | b2933 | 103 | 0.61 | PTS system, mannitol-specific enzyme II component |
| exuT | b3093 | 152 | 0.72 | ExuT |
| frwD | b3953 | 59 | 0.50 | PTS system fructose-like IIB component 2 |
| glpT | b2240 | 164 | 0.77 | sn-glycerol-3-phosphate permease |
| rbsD | b3748 | 157 | 0.74 | RbsD |
| yadI | b0129 | 58 | 0.50 | putative PTS enzyme II B component |
| yceE | b1053 | 5 | 0.36 | putative transport protein |
| yfbH | b2256 | 149 | 0.71 | hypothetical protein |
| ygbL | b2738 | 94 | 0.59 | putative epimerase/aldolase |
| yicK | b3659 | 132 | 0.66 | two-module transport protein |
| yihN | b3874 | 180 | 0.83 | putative resistance protein |
| yqaD | b2658 | 56 | 0.50 | hypothetical protein |
| ytfS | b4229 | 38 | 0.47 | YtfS |
| E: Amino acid transport and metabolism | | | | |
| argR | b3237 | 44 | 0.48 | ArgR |
| gltJ | b0654 | 54 | 0.50 | GltJ |
| ilvN | b3670 | 9 | 0.39 | acetolactate synthase I small subunit |
| selA | b3591 | 184 | 0.87 | selenocysteine synthase |
| yggP | b2932 | 11 | 0.40 | hypothetical protein |
| yigJ | b3823 | 20 | 0.42 | hypothetical protein |
| F: Nucleotide transport and metabolism | | | | |
| htrA | b0161 | 88 | 0.58 | deoxyguanosine triphosphate triphosphohydrolase |
| purN | b2500 | 115 | 0.63 | phosphoribosylglycinamide formyltransferase 1 |
| pyrE | b3642 | 72 | 0.53 | orotate phosphoribosyltransferase |
| H: Coenzyme metabolism | | | | |
| folX | b2303 | 81 | 0.56 | D-erythro-7,8-dihydroneopterin tri P epimerase |
| panD | b0131 | 120 | 0.64 | aspartate 1-decarboxylase |
| ribD | b0414 | 17 | 0.41 | pyrimidine deaminase |
| I: Lipid metabolism | | | | |
| idi | b2889 | 107 | 0.61 | hypothetical protein |
| pgpA | b0418 | 169 | 0.79 | phosphatidylglycerophosphatase |
| plsC | b3018 | 173 | 0.80 | 1-acyl-sn-glycerol-3-phosphate acyltransferase |
| psd | b4160 | 109 | 0.62 | phosphatidylserine decarboxylase |
| Q: Secondary metabolites biosynthesis, transport and catabolism | | | | |
| yciK | b1271 | 147 | 0.70 | putative oxidoreductase |
| ygfF | b2902 | 172 | 0.80 | putative oxidoreductase |
| Poorly characterized | | | | |
| R: General function prediction only | | | | |
| b0165 (f43) | b0165 | 76 | 0.54 | hypothetical protein |
| glpG | b3424 | 163 | 0.76 | GlpG |
| yceG | b1097 | 33 | 0.46 | putative thymidylate kinase |
| yfbT | b2293 | 27 | 0.44 | putative phosphatase |
| yhfV | b3379 | 36 | 0.46 | putative hydrolase |
| yhjW | b3546 | 179 | 0.83 | hypothetical protein |
| ynhA | b1679 | 141 | 0.68 | hypothetical protein |
| yrbA | b3190 | 55 | 0.50 | hypothetical protein |
| S: Function unknown | | | | |
| yajQ | b0426 | 89 | 0.58 | hypothetical protein |
| ybdF | b0579 | 136 | 0.67 | hypothetical protein |
| yceH | b1067 | 62 | 0.51 | hypothetical protein |
| ycfS | b1113 | 137 | 0.67 | hypothetical protein |
| ycfT | b1115 | 70 | 0.53 | hypothetical protein |
| yciF | b1258 | 105 | 0.61 | YciF |
| ydaS | b1357 | 60 | 0.50 | hypothetical protein |
| ydjR | b1742 | 150 | 0.71 | hypothetical protein |
| yebG | b1848 | 117 | 0.63 | hypothetical protein |
| yegJ | b2071 | 50 | 0.49 | hypothetical protein |
| yehR | b2123 | 19 | 0.42 | hypothetical protein |
| yehS | b2124 | 78 | 0.54 | hypothetical protein |
| yejL | b2187 | 96 | 0.59 | hypothetical protein |
| yfiH | b2593 | 90 | 0.58 | hypothetical protein |
| yhaI | b3104 | 47 | 0.48 | putative cytochrome |
| yhdT | b3257 | 113 | 0.63 | hypothetical protein |
| yheU | b3354 | 133 | 0.67 | hypothetical protein |
| yhhL | b3466 | 6 | 0.37 | hypothetical protein |
| yjdI | b4126 | 1 | 0.28 | hypothetical protein |
| yjfL | b4184 | 99 | 0.60 | hypothetical protein |
| yjjB | b4363 | 22 | 0.43 | hypothetical protein |
| yqhA | b3002 | 134 | 0.67 | hypothetical protein |
| No COG classification | | | | |
|  | b1788 | 4 | 0.35 | hypothetical protein |
| b0322 (o106) | b0322 | 29 | 0.44 | hypothetical protein |
| b0725 | b0725 | 135 | 0.67 | hypothetical protein |
| b0816 | b0816 | 30 | 0.45 | hypothetical protein |
| b2596 | b2596 | 74 | 0.54 | hypothetical protein |
| b3004 | b3004 | 104 | 0.61 | hypothetical protein |
| b3007 | b3007 | 139 | 0.68 | hypothetical protein |
| b3913 | b3913 | 118 | 0.64 |  |
| citE | b0616 | 144 | 0.69 | citrate lyase beta chain acyl lyase subunit |
| eaeH | b0297 | 112 | 0.63 | attaching and effacing protein |
| elaD | b2269 | 130 | 0.66 | putative sulfatase/phosphatase |
| gntU_2 | b3435 | 39 | 0.47 |  |
| ilvL | b3766 | 124 | 0.65 | ilvGEDA operon leader peptide |
| pinO | b3322 | 13 | 0.40 | PinO |
| pssR | b3763 | 177 | 0.82 |  |
| relF | b1562 | 121 | 0.65 | RelF |
| rfc | b2035 | 26 | 0.44 | O-antigen polymerase |
| trpL | b1265 | 75 | 0.54 | trp operon leader peptide |
| wcaD | b2056 | 12 | 0.40 | putative colanic acid polymerase |
| wcaM | b2043 | 161 | 0.75 | hypothetical protein |
| yacH | b0117 | 166 | 0.78 | putative membrane protein |
| yafZ | b0252 | 71 | 0.53 | hypothetical protein |
| ybeB | b0637 | 46 | 0.48 | hypothetical protein |
| ybfA | b0699 | 128 | 0.66 | hypothetical protein |
| ybfP | b0689 | 57 | 0.50 | putative pectinase |
| ybgO | b0716 | 52 | 0.49 | hypothetical protein |
| ybhT | b0762 | 7 | 0.37 | hypothetical protein |
| ybiX | b0804 | 110 | 0.62 | YbiX |
| ybjH | b0843 | 73 | 0.53 | hypothetical protein |
| yccU | b0965 | 174 | 0.81 | hypothetical protein |
| yceP | b1060 | 64 | 0.51 | hypothetical protein |
| ycfP | b1108 | 40 | 0.47 | hypothetical protein |
| ycfQ | b1111 | 45 | 0.48 | hypothetical protein |
| ydcD | b1457 | 114 | 0.63 | hypothetical protein |
| yddJ | b1470 | 67 | 0.52 | hypothetical protein |
| ydfP | b1553 | 48 | 0.49 | hypothetical protein |
| ydfZ | b1541 | 125 | 0.65 | hypothetical protein |
| ydhR | b1667 | 122 | 0.65 | hypothetical protein |
| yebW | b1837 | 63 | 0.51 | hypothetical protein |
| yecH | b1906 | 23 | 0.43 | hypothetical protein |
| yedD | b1928 | 86 | 0.56 | hypothetical protein |
| yedR | b1963 | 142 | 0.69 | hypothetical protein |
| yfcI | b2305 | 66 | 0.52 | hypothetical protein |
| yfdT | b2363 | 53 | 0.50 | hypothetical protein |
| yfjD | b2612 | 69 | 0.52 |  |
| yfjF | b2618 | 119 | 0.64 | hypothetical protein |
| ygaY | b2680 | 182 | 0.85 |  |
| ygbF | b2754 | 145 | 0.70 | hypothetical protein |
| ygcG | b2778 | 126 | 0.65 | hypothetical protein |
| ygeO | b2857 | 91 | 0.59 | hypothetical protein |
| yghR | b2984 | 116 | 0.63 | hypothetical protein |
| ygiA | b3036 | 65 | 0.52 | hypothetical protein |
| ygiQ | b3016 | 93 | 0.59 |  |
| yhbC | b3170 | 25 | 0.43 | hypothetical protein |
| yhfL | b3369 | 2 | 0.32 | hypothetical protein |
| yhfY | b3382 | 176 | 0.81 | hypothetical protein |
| yibG | b3596 | 24 | 0.43 | hypothetical protein |
| yigE | b3815 | 165 | 0.77 |  |
| yjbD | b4023 | 158 | 0.74 | hypothetical protein |
| yjeI | b4144 | 80 | 0.55 | hypothetical protein |
| yjiD | b4326 | 106 | 0.61 | hypothetical protein |
| yjiP | b4338 | 35 | 0.46 | hypothetical protein |
| ymcD | b0987 | 148 | 0.70 | hypothetical protein |
| yoaG | b1796 | 49 | 0.49 | hypothetical protein |
| ypfG | b2466 | 185 | 0.87 | hypothetical protein |
| yqeK | b2849 | 41 | 0.47 | hypothetical protein |
| yqgD | b2941 | 28 | 0.44 | hypothetical protein |
| yqhE | b3012 | 123 | 0.65 | hypothetical protein |
| yrbB | b3191 | 42 | 0.48 | hypothetical protein |
| yrhB | b3446 | 31 | 0.45 | hypothetical protein |

**a.** Gene names according to *E. coli* Colibri database (http://genolist.pasteur.fr/Colibri/).

**b.** Gene names according to Blattner nomenclature (http://www.genome.wisc.edu/sequencing/k12.htm#gen).

**c.** Rank position; 1 = most repressed gene in self-infected biofilm (C+C) versus non-infected monospecies commensal (C) biofilm.

**d.** Ratio of gene expression in *E. coli* MG1655 F’ + MG1655 F’ biofilm (C + C) versus gene expression in MG1655 F’ biofilm (C).

**e.** Function description according to the COG functional categories annotation system used by the NCBI (http://www.ncbi.nlm.nih.gov/COG).
