# Supplementary material for: Identification of Commensal Escherichia coli Genes Involved in Biofilm Resistance to Pathogen Colonization
Source: PLoS One. 2013 May 7;8(5):e61628. doi: 10.1371/journal.pone.0061628 (PMC3646849; doi:10.1371/journal.pone.0061628)
Supplement: Table S4 — Genes induced upon colonization by exogenous pathogen (C+P). (DOCX) [file pone.0061628.s006.docx]

**Table S4:** Genes induced upon colonization by exogenous pathogen (C+P)

| **Gene name** | | | | | | | | C + P /C^c^ | | | | | | | C + P / **C + C^d^** | | | | | | | **Function-description^f^** | |  |  |
| --- | --- | --- | --- | --- | --- | --- | --- | --- | --- | --- | --- | --- | --- | --- | --- | --- | --- | --- | --- | --- | --- | --- | --- | --- | --- |
| **a** | | | **b** | | | | | **Rank^e^** | | | **Ratio** | | | | **Rank^e^** | | | | **Ratio** | | |  |  |  |  |
| **Information storage and processing** | | | | | | | | | | | | | | | | | | | | | | | |  |  |
| *J: Translation, ribosomal structure and biogenesis* | | | | | | | | | | | | | | | | | | | | | | | |  |  |
|  | *argS* | | b1876 | | | 61 | | | | 1.44 | | | |  | | | | | |  | arginine tRNA synthetase | |  |  |  |
|  | *rsmB* | | b3289 | | | | 141 | | | | | 1.31 | |  | | | | | |  | 16S rRNA m^5^C967 methytransferase | |  |  |  |
|  | *fusA* | | b3340 | | | | 117 | | | | | 1.36 | |  | | | | | |  | GTP-binding protein chain elongation factor EF-G | |  |  |  |
|  | *lysU* | | b4129 | | | | 12 | | | | | 1.74 | | 89 | | | | | | 1.39 | lysine tRNA synthetase | |  |  |  |
|  | *rimL* | | b1427 | | | | 205 | | | | | 1.22 | |  | | | | | |  | acetyl transferase | |  |  |  |
|  | *rnd* | | b1804 | | | | 198 | | | | | 1.24 | |  | | | | | |  | RNase D | |  |  |  |
|  | *bamE* | | b2617 | | | |  | | | | |  | | 66 | | | | | | 1.60 | small membrane protein A | |  |  |  |
|  | *tsf* | | b0170 | | | |  | | | | |  | | 5 | | | | | | 2.53 | protein chain elongation factor EF-Ts | |  |  |  |
|  | *yabO* | | b0058 | | | | 206 | | | | | 1.22 | | 94 | | | | | | 1.32 | pseudouridylate synthase | |  |  |  |
| K: Transcription | | | | | | | | | | | | | | | | | | | | | | | |  |  |
|  | *abgR* | | | b1339 | | | | |  | | | |  | 91 | | | | | 1.36 | | putative transcriptional regulator LYSR-type | | | |  |
|  | *asnC* | | b3743 | | | | |  | | |  | | | | 51 | | 1.74 | | | | | AsnC | |  |  |
|  | *bglG* | | b3723 | | | | |  | | |  | | | | 71 | | 1.52 | | | | | BglG | |  |  |
|  | *cadC* | | b4133 | | | | | 130 | | | 1.34 | | | |  | |  | | | | | CadC | |  |  |
|  | *cspF******** | | b1558 | | | | |  | | |  | | | | 32 | | 1.99 | | | | | CspF, cold shock protein; Qin propahge | |  |  |
|  | *dicA* | | b1570 | | | | |  | | |  | | | | 22 | | 2.09 | | | | | DicA | |  |  |
|  | *envY* | | b0566 | | | | |  | | |  | | | | 25 | | 2.04 | | | | | envelope protein | |  |  |
|  | *fecI* | | b4293 | | | | |  | | |  | | | | 96 | | 1.31 | | | | | RNA polymerase, sigma 19 factor | |  |  |
|  | *hepA* | | b0059 | | | | | 42 | | | 1.49 | | | |  | |  | | | | | putative ATP-dependent RNA helicase | |  |  |
|  | *dgsA* | | b1594 | | | | | 186 | | | 1.25 | | | |  | |  | | | | | putative NAGC-like transcriptional regulator | |  |  |
|  | *rcsA******** | | b1951 | | | | |  | | |  | | | | 39 | | 1.88 | | | | | RcsA | |  |  |
|  | *rnk* | | b0610 | | | | |  | | |  | | | | 104 | | 1.21 | | | | | regulator of nucleoside diphosphate kinase | |  |  |
|  | *rpoN* | | b3202 | | | | | 217 | | | 1.18 | | | |  | |  | | | | | RpoN | |  |  |
|  | *rsd* | | b3995 | | | | | 122 | | | 1.35 | | | |  | |  | | | | | putative anti-sigma factor | |  |  |
|  | *uxuR* | | b4324 | | | | | 137 | | | 1.33 | | | |  | |  | | | | | UxuR | |  |  |
|  | *yagI* | | b0272 | | | | | 65 | | | 1.43 | | | |  | |  | | | | | putative regulator, CP4-6 prophage | |  |  |
|  | *yahB* | | b0316 | | | | | 35 | | | 1.53 | | | |  | |  | | | | | putative transcriptional regulator LYSR-type | |  |  |
|  | *ybcM* | | b0546 | | | | | 134 | | | 1.33 | | | |  | |  | | | | | putative ARAC-type regulatory protein | |  |  |
|  | *ybdO* | | b0603 | | | | | 69 | | | 1.43 | | | |  | |  | | | | | putative transcriptional regulator LYSR-type | |  |  |
|  | *ydcR* | | b1439 | | | | | 76 | | | 1.42 | | | |  | |  | | | | | hypothetical protein | |  |  |
|  | *mlrA* | | b2127 | | | | | 219 | | | 1.16 | | | |  | |  | | | | | MlrA; curli production transcriptional regulator | |  |  |
|  | *iscR* | | b2531 | | | | | 197 | | | 1.24 | | | |  | |  | | | | | Iron-sulfur cluster regulator | |  |  |
|  | *ygbI* | | b2735 | | | | | 208 | | | 1.21 | | | |  | |  | | | | | putative DEOR-type transcriptional regulator | |  |  |
|  | *gadW* | | b3515 | | | | |  | | |  | | | | 50 | | 1.75 | | | | | Glutamic acid decarboxylase transcriptional factor | |  |  |
|  | *yjcT* | | b4084 | | | | | 142 | | | 1.31 | | | |  | |  | | | | | putative NAGC-like transcriptional regulator | |  |  |
| L: DNA replication, recombination and repair | | | | | | | | | | | | | | | | | | | | | | | |  |  |
|  | *InsoO-1* | | b0257 | | | | | 154 | | | 1.30 | | | |  | |  | | | | | putative transposase | | | |
|  | *dbpA* | | b1343 | | | | | 83 | | | 1.40 | | | |  | |  | | | | | ATP-dependent RNA helicase | |  |  |
|  | *dcm* | | b1961 | | | | | 169 | | | 1.28 | | | |  | |  | | | | | DNA cytosine methylase | |  |  |
|  | *dnaB* | | b4052 | | | | | 181 | | | 1.26 | | | |  | |  | | | | | replicative DNA helicase | |  |  |
|  | *pinR* | | b1374 | | | | | 64 | | | 1.43 | | | |  | |  | | | | | putative transposon resolvase | |  |  |
|  | *priB* | | b4201 | | | | | 135 | | | 1.33 | | | |  | |  | | | | | primosomal replication protein N | |  |  |
|  | *sbcB* | | b2011 | | | | |  | | |  | | | | 31 | | 2.00 | | | | | exonuclease I | |  |  |
|  | *srmB* | | b2576 | | | | |  | | |  | | | | 101 | | 1.28 | | | | | ATP-dependent RNA helicase | |  |  |
|  | *yeeS* | | b2002 | | | | |  | | |  | | | | 60 | | 1.64 | | | | | putative DNA repair protein, RADC family | |  |  |
| Cellular processes | | | | | | | | | | | | | | | | | | | | | | | |  |  |
| ***O: Posttranslational modification, protein turnover,chaperones*** | | | | | | | | | | | | | | | | | | | | | | | |  |  |
|  | *ccmB* | | | | b2200 | | | |  | | |  | | | 82 | | 1.45 | | | | | heme exporter protein B | | | |
|  | *dsbG* | | b0604 | | | | | 44 | | | 1.49 | | | |  | |  | | | | | thiol:disulfide interchange protein | |  |  |
|  | *hscA* | | b2526 | | | | | 127 | | | 1.34 | | | |  | |  | | | | | HscA | |  |  |
|  | *htpG* | | b0473 | | | | | 171 | | | 1.28 | | | |  | |  | | | | | chaperone Hsp90 | |  |  |
|  | *sppA******** | | b1766 | | | | | 6 | | | 1.91 | | | | 38 | | 1.92 | | | | | protease IV | |  |  |
| M: Cell envelope biogenesis, outer membrane | | | | | | | | | | | | | | | | | | | | | | | |  |  |
|  | *aefA* | | | | b0465 | | | | 176 | | | | 1.26 | | |  | | |  | | | putative alpha helix protein | | | |
|  | *ddlA* | | b0381 | | | | |  | | |  | | | | 49 | | 1.75 | | | | | D-alanine-D-alanine ligase A | |  |  |
|  | *dniR* | | b0211 | | | | | 79 | | | 1.41 | | | |  | |  | | | | | cytochrome c552 | |  |  |
|  | *lepA* | | b2569 | | | | | 183 | | | 1.26 | | | |  | |  | | | | | GTP-binding elongation factor | |  |  |
|  | *mepA* | | b2328 | | | | | 163 | | | 1.29 | | | |  | |  | | | | | murein DD-endopeptidase | |  |  |
|  | *rfaG* | | b3631 | | | | | 92 | | | 1.39 | | | |  | |  | | | | | glucosyltransferase I | |  |  |
|  | *rhsD* | | b0497 | | | | |  | | |  | | | | 12 | | 2.26 | | | | | RhsD | |  |  |
|  | *rlpA* | | b0633 | | | | | 194 | | | 1.24 | | | |  | |  | | | | | RlpA | |  |  |
|  | *vacJ* | | b2346 | | | | | 91 | | | 1.39 | | | |  | |  | | | | | lipoprotein precursor | |  |  |
|  | *wcaJ* | | b2047 | | | | |  | | |  | | | | 85 | | 1.42 | | | | | putative colanic acid biosynthesis UDP-glucose lipid carrier transferase | |  |  |
|  | *yaeT******** | | b0177 | | | | | 24 | | | | 1.58 | |  | | | | |  | | hypothetical protein | | | | |
|  | *yefA* | | b2053 | | | | | 182 | | | 1.26 | | | |  | |  | | | | | GDP-D-mannose dehydratase | |  |  |
|  | *yrbM* | | b3208 | | | | | 157 | | | 1.30 | | | |  | |  | | | | | putative peptidoglycan enzyme | |  |  |
| ***N: Cell motility and secretion*** | | | | | | | | | | | | | | | | | | | | | | | |  |  |
|  | *flgB* | | | b1073 | | | 20 | | | | | 1.59 | |  | | | |  | | | FlgB | | | | |
|  | *flgJ* | | b1081 | | | | | 190 | | | 1.25 | | | |  | |  | | | | | FlgJ | |  |  |
|  | *flhA* | | b1879 | | | | | 56 | | | 1.45 | | | |  | |  | | | | | FlhA | |  |  |
|  | *fliF* | | b1938 | | | | |  | | |  | | | | 72 | | 1.52 | | | | | FliF | |  |  |
|  | *fliP* | | b1948 | | | | |  | | |  | | | | 47 | | 1.78 | | | | | FliP | |  |  |
|  | *sfmH* | | b0533 | | | | | 47 | | | 1.49 | | | | 16 | | 2.18 | | | | | involved in fimbrial asembly | |  |  |
|  | *tar* | | b1886 | | | | | 131 | | | 1.34 | | | |  | |  | | | | | methyl-accepting chemotaxis protein II | |  |  |
|  | *ycbQ******** | | b0938 | | | | | 26 | | | 1.57 | | | |  | |  | | | | | putative fimbrial-like protein | |  |  |
|  | *ycbR* | | b0939 | | | | | 124 | | | 1.35 | | | |  | |  | | | | | putative chaperone | |  |  |
|  | *ydeT* | | b1505 | | | | | 98 | | | 1.38 | | | |  | |  | | | | | putative outer membrane protein | |  |  |
| ***P: Inorganic ion transport and metabolism*** | | | | | | | | | | | | | | | | | | | | | | | |  |  |
|  | *cysQ* | | | b4214 | | | 93 | | | | | 1.39 | |  | | | | |  | | CysQ | | | | |
|  | *fepD* | | b0590 | | | | | 104 | | | 1.37 | | | |  | |  | | | | | FepD | |  |  |
|  | *fhuE* | | b1102 | | | | | 132 | | | 1.34 | | | |  | |  | | | | | outer membrane receptor for ferric iron uptake | |  |  |
|  | *narU* | | b1469 | | | | | 89 | | | 1.39 | | | |  | |  | | | | | nitrite extrusion protein 2 | |  |  |
|  | *phnE* | | b4103 | | | | |  | | |  | | | | 10 | | 2.30 | | | | | hypothetical protein | |  |  |
|  | *phnH* | | b4100 | | | | | 187 | | | 1.25 | | | |  | |  | | | | | PhnH | |  |  |
|  | *phnM* | | b4095 | | | | | 172 | | | 1.27 | | | |  | |  | | | | | PhnM | |  |  |
|  | *pitA* | | b3493 | | | | | 109 | | | 1.37 | | | |  | |  | | | | | low-affinity phosphate transport | |  |  |
|  | *pitB* | | b2987 | | | | | 110 | | | 1.37 | | | |  | |  | | | | | low-affinity phosphate transport | |  |  |
|  | *tauA* | | b0365 | | | | | 38 | | | 1.51 | | | |  | |  | | | | | TauA | |  |  |
|  | *tauC* | | b0367 | | | | | 178 | | | 1.26 | | | |  | |  | | | | | TauC | |  |  |
|  | *tehA* | | b1429 | | | | | 95 | | | 1.38 | | | |  | |  | | | | | TehA | |  |  |
|  | *trkG* | | b1363 | | | | | 87 | | | 1.40 | | | |  | |  | | | | | trk system potassium uptake | |  |  |
|  | *ybiL* | | b0805 | | | | | 28 | | | 1.56 | | | |  | |  | | | | | putative outer membrane receptor for iron transport | |  |  |
|  | *ydeN* | | b1498 | | | | |  | | |  | | | | 44 | | 1.82 | | | | | putative sulfatase | |  |  |
|  | *yfbS* | | b2292 | | | | | 86 | | | 1.40 | | | |  | |  | | | | | putative transport protein | |  |  |
|  | *yfdC* | | b2347 | | | | |  | | |  | | | | 84 | | 1.43 | | | | | putative transport | |  |  |
|  | *yiiP* | | b3915 | | | | | 192 | | | 1.25 | | | |  | |  | | | | | putative transport system permease protein | |  |  |
| ***T: Signal transduction mechanisms*** | | | | | | | | | | | | | | | | | | | | | | | |  |  |
|  | *baeS* | | | b2078 | | | 204 | | | | | 1.22 | |  | | | | |  | | BaeS | | | | |
|  | *cpxA* | | b3911 | | | | | 59 | | | 1.44 | | | |  | |  | | | | | CpxA | |  |  |
|  | *creC* | | b4399 | | | | | 147 | | | 1.31 | | | |  | |  | | | | | CreC | |  |  |
|  | *dpiB* | | b0619 | | | | | 113 | | | 1.36 | | | |  | |  | | | | | putative sensor-type protein | |  |  |
|  | *fnr* | | b1334 | | | | | 84 | | | 1.40 | | | | 93 | | 1.35 | | | | | Fnr | |  |  |
|  | *phoH* | | b1020 | | | | | 90 | | | 1.39 | | | |  | |  | | | | | PhoH | |  |  |
|  | *wzb* | | b2061 | | | | |  | | |  | | | | 79 | | 1.48 | | | | | putative protein-tyrosine-phosphatase | |  |  |
|  | *yaiC* | | b0385 | | | | | 102 | | | 1.37 | | | |  | |  | | | | | hypothetical protein | |  |  |
|  | *ycdT* | | b1025 | | | | | 50 | | | 1.48 | | | | 55 | | 1.72 | | | | | hypothetical protein | |  |  |
|  | *ygiY* | | b3026 | | | | | 107 | | | 1.37 | | | |  | |  | | | | | quorum sensing regulator C | |  |  |
|  | *yjiY******** | | b4354 | | | | | 34 | | | 1.53 | | | |  | |  | | | | | putative carbon starvation protein | |  |  |
|  | *yliE******** | | b0833 | | | | |  | | |  | | | | 34 | | 1.99 | | | | | hypothetical protein | |  |  |
| U: Intracellular trafficking, secretion and vesicular transport | | | | | | | | | | | | | | | | | | | | | | | |  |  |
|  | *secE* | | | b3981 | | | 158 | | | | | 1.30 | | 95 | | | | 1.32 | | | preprotein translocase | | | | |
|  | *ychE* | | b1242 | | | | | 68 | | | 1.43 | | | |  | |  | | | | | putative channel protein | |  |  |
|  | *ydeB* | | b1529 | | | | |  | | |  | | | | 76 | | 1.51 | | | | | hypothetical protein | |  |  |
| V: Defense mechanisms | | | | | | | | | | | | | | | | | | | | | | | |  |  |
|  | *yiaV* | | | b3586 | | | 40 | | | | | 1.50 | |  | | | | |  | | putative membrane protein | | | | |
|  | *yibH* | | b3597 | | | | |  | | |  | | | | 70 | | 1.53 | | | | | putative membrane protein | |  |  |
|  | *yjcR* | | b4082 | | | | | 11 | | | 1.74 | | | |  | |  | | | | | putative membrane protein | |  |  |
| **Metabolism** | | | | | | | | | | | | | | | | | | | | | | | |  |  |
| ***C: Energy production and conversion*** | | | | | | | | | | | | | | | | | | | | | | | |  |  |
|  | *acnB* | | | b0118 | | | 139 | | | | | 1.32 | |  | | | | |  | | aconitate hydrase B | | | | |
|  | *adhC* | | b0356 | | | | | 188 | | | 1.25 | | | |  | |  | | | | | alcohol dehydrogenase class III | |  |  |
|  | *aldA* | | b1415 | | | | | 15 | | | 1.65 | | | |  | |  | | | | | aldehyde dehydrogenase, NAD-linked | |  |  |
|  | *cydC* | | b0886 | | | | | 191 | | | 1.25 | | | |  | |  | | | | | ATP-binding component of cytochrome-related transport | |  |  |
|  | *cydD* | | b0887 | | | | | 180 | | | 1.26 | | | |  | |  | | | | | ATP-binding component of cytochrome-related transport | |  |  |
|  | *eutE* | | b2455 | | | | | 13 | | | 1.71 | | | |  | |  | | | | | EutE | |  |  |
|  | *fdnG* | | b1474 | | | | | 112 | | | 1.36 | | | |  | |  | | | | | formate dehydrogenase-N, nitrate-inducible, alpha subunit | |  |  |
|  | *fdrA* | | b0518 | | | | | 114 | | | 1.36 | | | |  | |  | | | | | FdrA | |  |  |
|  | *fixA* | | b0041 | | | | | 120 | | | 1.35 | | | |  | |  | | | | | putative flavoprotein subunit | |  |  |
|  | *fixB* | | b0042 | | | | | 167 | | | 1.28 | | | |  | |  | | | | | putative flavoprotein subunit | |  |  |
|  | *sfcA* | | b1479 | | | | | 155 | | | 1.30 | | | |  | |  | | | | | NAD-linked malate dehydrogenase | |  |  |
|  | *ydgN* | | b1629 | | | | | 164 | | | 1.29 | | | |  | |  | | | | | putative membrane protein | |  |  |
|  | *ydhY* | | b1674 | | | | | 81 | | | 1.41 | | | | 102 | | 1.27 | | | | | putative oxidoreductase Fe-S subunit | |  |  |
|  | *yfdE* | | b2371 | | | | |  | | |  | | | | 37 | | 1.94 | | | | | hypothetical protein | |  |  |
|  | *yfhN* | | b2529 | | | | |  | | |  | | | | 77 | | 1.48 | | | | | hypothetical protein | |  |  |
|  | *yiaY* | | b3589 | | | | |  | | |  | | | | 46 | | 1.79 | | | | | putative oxidoreductase | |  |  |
|  | *ynfE* | | b1587 | | | | | 62 | | | 1.43 | | | |  | |  | | | | | putative oxidoreductase major subunit | |  |  |
| G: Carbohydrate transport and metabolism | | | | | | | | | | | | | | | | | | | | | | | |  |  |
|  | *agaB* | | | b3138 | | | | |  | | | |  | 61 | | | | 1.63 | | | PTS system, cytoplasmic, N-acetylgalactosamine-specific IIB component 1 | | | | |
|  | *agaD* | | b3140 | | | | |  | | |  | | | | 45 | | 1.80 | | | | | PTS system, N-acetylglucosamine enzyme IID component 1 | |  |  |
|  | *agaI******** | | b3141 | | | | | 7 | | | 1.89 | | | | 9 | | 2.37 | | | | | putative galactosamine-6-phosphate isomerase | |  |  |
|  | *agaY* | | b3137 | | | | | 70 | | | 1.43 | | | | 59 | | 1.67 | | | | | tagatose-bisphosphate aldolase 2 | |  |  |
|  | *araF* | | b1901 | | | | | 32 | | | 1.55 | | | | 83 | | 1.43 | | | | | L-arabinose-binding periplasmic protein | |  |  |
|  | *araG* | | b1900 | | | | | 29 | | | 1.56 | | | |  | |  | | | | | ATP-binding component of high-affinity L-arabinose transport system | |  |  |
|  | *bglF* | | b3722 | | | | | 72 | | | 1.42 | | | |  | |  | | | | | PTS system beta-glucosides | |  |  |
|  | *celA* | | b1738 | | | | |  | | |  | | | | 29 | | 2.00 | | | | | CelA | |  |  |
|  | *celC* | | b1736 | | | | | 207 | | | 1.22 | | | |  | |  | | | | | CelC | |  |  |
|  | *dsdX* | | b2365 | | | | | 74 | | | 1.42 | | | |  | |  | | | | | transport system permease | |  |  |
|  | *frvR* | | b3897 | | | | | 184 | | | 1.25 | | | |  | |  | | | | | putative frv operon regulatory protein | |  |  |
|  | *frvX* | | b3898 | | | | | 220 | | | 1.16 | | | | 107 | | 1.18 | | | | | frv operon protein | |  |  |
|  | *fucI* | | b2802 | | | | |  | | |  | | | | 108 | | 1.15 | | | | | L-fucose isomerase | |  |  |
|  | *gsk* | | b0477 | | | | | 202 | | | 1.23 | | | |  | |  | | | | | inosine-guanosine kinase | |  |  |
|  | *hrsA* | | b0731 | | | | | 94 | | | 1.39 | | | |  | |  | | | | | HrsA | |  |  |
|  | *kduI******** | | b2843 | | | | | 2 | | | 2.88 | | | | 3 | | 2.57 | | | | | homolog of pectin degrading enzyme 5-keto 4-deoxyuronate isomerase | |  |  |
|  | *mtlA* | | b3599 | | | | | 196 | | | 1.24 | | | |  | |  | | | | | PTS system, mannitol-specific enzyme IIABC components | |  |  |
|  | *mtlD* | | b3600 | | | | | 170 | | | 1.28 | | | |  | |  | | | | | mannitol-1-phosphate dehydrogenase | |  |  |
|  | *otsA* | | b1896 | | | | | 96 | | | 1.38 | | | |  | |  | | | | | trehalose-6-phosphate synthase | |  |  |
|  | *ppsA* | | b1702 | | | | | 75 | | | 1.42 | | | |  | |  | | | | | phosphoenolpyruvate synthase | |  |  |
|  | *ptsA* | | b3947 | | | | | 150 | | | 1.30 | | | |  | |  | | | | | PEP-protein phosphotransferase system enzyme I | |  |  |
|  | *rhaA* | | b3903 | | | | | 216 | | | 1.18 | | | |  | |  | | | | | L-rhamnose isomerase | |  |  |
|  | *rhaD* | | b3902 | | | | | 148 | | | 1.31 | | | |  | |  | | | | | rhamnulose-phosphate aldolase | |  |  |
|  | *uidB* | | b1616 | | | | | 218 | | | 1.16 | | | |  | |  | | | | | glucuronide permease | |  |  |
|  | *xylF* | | b3566 | | | | | 54 | | | 1.46 | | | |  | |  | | | | | xylose binding protein transport system | |  |  |
|  | *yadI* | | b0129 | | | | |  | | |  | | | | 19 | | 2.15 | | | | | putative PTS enzyme II B component | |  |  |
|  | *yagH* | | b0271 | | | | |  | | |  | | | | 67 | | 1.59 | | | | | putative beta-xylosidase | |  |  |
|  | *ybgB* | | b0732 | | | | | 129 | | | 1.34 | | | |  | |  | | | | | putative sugar hydrolase | |  |  |
|  | *ydfI* | | b1542 | | | | | 25 | | | 1.57 | | | |  | |  | | | | | putative oxidoreductase | |  |  |
|  | *ydfJ* | | b1543 | | | | | 126 | | | 1.35 | | | |  | |  | | | | | putative transport protein | |  |  |
|  | *ydhC* | | b1660 | | | | |  | | |  | | | | 90 | | 1.38 | | | | | putative transport protein | |  |  |
|  | *ydjK* | | b1775 | | | | | 161 | | | 1.29 | | | |  | |  | | | | | putative transport protein | |  |  |
|  | *yegT* | | b2098 | | | | | 118 | | | 1.36 | | | |  | |  | | | | | putative nucleoside permease protein | |  |  |
|  | *yeiC* | | b2166 | | | | | 152 | | | 1.30 | | | |  | |  | | | | | putative kinase | |  |  |
|  | *yeiQ* | | b2172 | | | | | 105 | | | 1.37 | | | |  | |  | | | | | putative oxidoreductase | |  |  |
|  | *yfbH* | | b2256 | | | | |  | | |  | | | | 81 | | 1.45 | | | | | hypothetical protein | |  |  |
|  | *ygbN* | | b2740 | | | | | 214 | | | 1.18 | | | |  | |  | | | | | putative transport protein | |  |  |
|  | *yhaU* | | b3127 | | | | | 31 | | | 1.56 | | | |  | |  | | | | | putative transport protein | |  |  |
|  | *yhfQ* | | b3374 | | | | |  | | |  | | | | 106 | | 1.20 | | | | | putative kinase | |  |  |
|  | *yicK* | | b3659 | | | | |  | | |  | | | | 69 | | 1.54 | | | | | two-module transport protein | |  |  |
|  | *yieC* | | b3720 | | | | | 125 | | | 1.35 | | | |  | |  | | | | | putative receptor protein | |  |  |
|  | *yjcW* | | b4087 | | | | | 146 | | | 1.31 | | | |  | |  | | | | | putative ATP-binding component of a transport system | |  |  |
|  | *yjhF* | | b4296 | | | | | 174 | | | 1.27 | | | |  | |  | | | | | putative transport system permease | |  |  |
|  | *ypdD* | | b2383 | | | | | 21 | | | 1.58 | | | |  | |  | | | | | putative PTS system enzyme IIA component, enzyme I | |  |  |
|  | *yqaD* | | b2658 | | | | |  | | |  | | | | 21 | | 2.09 | | | | | hypothetical protein | |  |  |
|  | *yqcE* | | b2775 | | | | |  | | |  | | | | 48 | | 1.76 | | | | | putative transport protein | |  |  |
| ***E: Amino acid transport and metabolism*** | | | | | | | | | | | | | | | | | | | | | | | |  |  |
|  | *argE* | | | b3957 | | | 199 | | | | | 1.24 | |  | | | | |  | | acetylornithine deacetylase | | | | |
|  | *argG* | | b3172 | | | | | 160 | | | 1.29 | | | |  | |  | | | | | argininosuccinate synthetase | |  |  |
|  | *argI* | | b4254 | | | | | 4 | | | 2.03 | | | |  | |  | | | | | ornithine carbamoyltransferase 1 | |  |  |
|  | *argR* | | b3237 | | | | |  | | |  | | | | 30 | | 2.00 | | | | | ArgR | |  |  |
|  | *aroD* | | b1693 | | | | | 10 | | | 1.75 | | | | 52 | | 1.74 | | | | | 3-dehydroquinate dehydratase | |  |  |
|  | *betA* | | b0311 | | | | | 23 | | | 1.58 | | | |  | |  | | | | | choline dehydrogenase | |  |  |
|  | *cadB* | | b4132 | | | | | 133 | | | 1.34 | | | |  | |  | | | | | transport of lysine/cadaverine | |  |  |
|  | *carA* | | b0032 | | | | | 211 | | | 1.21 | | | | 105 | | 1.20 | | | | | carbamoyl-phosphate synthetase | |  |  |
|  | *dapA* | | b2478 | | | | | 151 | | | 1.30 | | | |  | |  | | | | | dihydrodipicolinate synthase | |  |  |
|  | *dsdA* | | b2366 | | | | | 8 | | | 1.81 | | | | 35 | | 1.98 | | | | | D-serine dehydratase | |  |  |
|  | *gatD* | | b2091 | | | | | 212 | | | 1.19 | | | |  | |  | | | | | galactitol-1-phosphate dehydrogenase | |  |  |
|  | *ilvC* | | b3774 | | | | | 106 | | | 1.37 | | | |  | |  | | | | | ketol-acid reductoisomerase | |  |  |
|  | *leuA* | | b0074 | | | | |  | | |  | | | | 80 | | 1.47 | | | | | 2-isopropylmalate synthase | |  |  |
|  | *metB* | | b3939 | | | | | 123 | | | 1.35 | | | |  | |  | | | | | cystathionine gamma-synthase | |  |  |
|  | *ptrB* | | b1845 | | | | | 52 | | | 1.47 | | | |  | |  | | | | | protease II | |  |  |
|  | *sdaB* | | b2797 | | | | | 140 | | | 1.32 | | | |  | |  | | | | | L-serine dehydratase | |  |  |
|  | *solA* | | b1059 | | | | | 144 | | | 1.31 | | | |  | |  | | | | | sarcosine oxidase-like protein | |  |  |
|  | *speF* | | b0693 | | | | |  | | |  | | | | 26 | | 2.03 | | | | | ornithine decarboxylase isozyme | |  |  |
|  | *tyrA* | | b2600 | | | | | 43 | | | 1.49 | | | |  | |  | | | | | chorismate mutase-T | |  |  |
|  | *ycaM* | | b0899 | | | | | 27 | | | 1.57 | | | |  | |  | | | | | putative transport | |  |  |
|  | *ydjJ* | | b1774 | | | | | 53 | | | 1.46 | | | |  | |  | | | | | putative oxidoreductase | |  |  |
|  | *yecC* | | b1917 | | | | |  | | |  | | | | 40 | | 1.88 | | | | | putative ATP-binding component of a transport system | |  |  |
|  | *yeiT* | | b2146 | | | | | 46 | | | 1.49 | | | |  | |  | | | | | putative oxidoreductase | |  |  |
|  | *yfbQ* | | b2290 | | | | |  | | |  | | | | 17 | | 2.18 | | | | | putative aminotransferase | |  |  |
|  | *yfdU* | | | b2373 | | | | |  | | | |  | 58 | | | | 1.68 | | | hypothetical protein | | | | |
|  | *yhfM* | | b3370 | | | | |  | | |  | | | | 68 | | 1.54 | | | | | putative amino acid/amine transport protein | |  |  |
|  | *yjdL* | | b4130 | | | | | 14 | | | 1.66 | | | |  | |  | | | | | putative peptide transporter | |  |  |
|  | *yliD* | | b0832 | | | | | 156 | | | 1.30 | | | |  | |  | | | | | putative transport system permease protein | |  |  |
| ***F: Nucleotide transport and metabolism*** | | | | | | | | | | | | | | | | | | | | | | | |  |  |
|  | *cpdB* | | | b4213 | | | 213 | | | | | 1.18 | |  | | | | |  | | 2',3'-cyclic-nucleotide 2'-phosphodiesterase | | | | |
|  | *htrA* | | b0161 | | | | |  | | |  | | | | 13 | | 2.25 | | | | | deoxyguanosine triphosphate triphosphohydrolase | |  |  |
|  | *nupC* | | b2393 | | | | | 100 | | | 1.38 | | | |  | |  | | | | | NupC | |  |  |
|  | *purB* | | b1131 | | | | | 30 | | | 1.56 | | | |  | |  | | | | | adenylosuccinate lyase | |  |  |
|  | *pyrD* | | b0945 | | | | | 39 | | | 1.50 | | | |  | |  | | | | | dihydro-orotate dehydrogenase | |  |  |
|  | *pyrF* | | b1281 | | | | | 5 | | | 1.93 | | | |  | |  | | | | | orotidine-5'-phosphate decarboxylase | |  |  |
|  | *yaaF* | | b0030 | | | | | 99 | | | 1.38 | | | |  | |  | | | | | hypothetical protein | |  |  |
|  | *yicE* | | b3654 | | | | |  | | |  | | | | 92 | | 1.36 | | | | | putative transport protein | |  |  |
| ***H: Coenzyme metabolism*** | | | | | | | | | | | | | | | | | | | | | | | |  |  |
|  | *btuB* | | | b3966 | | | 115 | | | | | 1.36 | |  | | | | |  | | BtuB | | | | |
|  | *coaA* | | b3974 | | | | | 128 | | | 1.34 | | | |  | |  | | | | | pantothenate kinase | |  |  |
|  | *moaC* | | b0783 | | | | | 57 | | | 1.45 | | | |  | |  | | | | | molybdopterin biosynthesis protein C | |  |  |
|  | *panD* | | b0131 | | | | |  | | |  | | | | 75 | | 1.51 | | | | | aspartate 1-decarboxylase | |  |  |
|  | *ydaH* | | b1336 | | | | | 19 | | | 1.59 | | | |  | |  | | | | | putative pump protein | |  |  |
| ***I: Lipid metabolism*** | | | | | | | | | | | | | | | | | | | | | | | |  |  |
|  | *caiA* | | | b0039 | | | 185 | | | | | 1.25 | |  | | | | |  | | putative carnitine operon oxidoreductase | | | | |
|  | *fadL* | | b2344 | | | | |  | | |  | | | | 73 | | 1.52 | | | | | FadL | |  |  |
|  | *yafH* | | b0221 | | | | | 215 | | | 1.18 | | | |  | |  | | | | | putative acyl-CoA dehydrogenase | |  |  |
|  | *ydiF* | | b1694 | | | | | 189 | | | 1.25 | | | |  | |  | | | | | hypothetical protein | |  |  |
| ***Q: Secondary metabolite biosynthesis. transport and catabolism*** | | | | | | | | | | | | | | | | | | | | | | | |  |  |
|  | *tynA* | | | b1386 | | | 179 | | | | | 1.26 | |  | | | | |  | | copper amine oxidase | | | | |
|  | *yafS* | | b0213 | | | | |  | | |  | | | | 23 | | 2.09 | | | | | hypothetical protein | |  |  |
|  | *ycgM* | | b1180 | | | | | 82 | | | 1.41 | | | |  | |  | | | | | putative isomerase | |  |  |
| **Poorly characterized** | | | | | | | | | | | | | | | | | | | | | | | |  |  |
| ***R: General function prediction only*** | | | | | | | | | | | | | | | | | | | | | | | |  |  |
|  | *elaA* | | | b2267 | | |  | | | | |  | | 78 | | | | 1.48 | | | hypothetical protein | | | | |
|  | *hipA* | | b1507 | | | | | 48 | | | 1.48 | | | |  | |  | | | | | HipA | |  |  |
|  | *hybF* | | b2991 | | | | |  | | |  | | | | 43 | | 1.85 | | | | | HybF | |  |  |
|  | *stpA* | | b2669 | | | | | 168 | | | 1.28 | | | | 86 | | 1.42 | | | | | StpA | |  |  |
|  | *uup* | | b0949 | | | | | 119 | | | 1.36 | | | |  | |  | | | | | putative ATP-binding component of a transport system | |  |  |
|  | *ybdN* | | b0602 | | | | | 162 | | | 1.29 | | | |  | |  | | | | | hypothetical protein | |  |  |
|  | *ybgL* | | b0713 | | | | | 22 | | | 1.58 | | | |  | |  | | | | | putative lactam utilization protein | |  |  |
|  | *ybiP* | | b0815 | | | | |  | | |  | | | | 87 | | 1.40 | | | | | hypothetical protein | |  |  |
|  | *yeaE* | | b1781 | | | | | 209 | | | 1.21 | | | |  | |  | | | | | putative aldehyde reductase | |  |  |
|  | *yegI* | | b2070 | | | | | 71 | | | 1.43 | | | | 97 | | 1.29 | | | | | putative chaperonin | |  |  |
|  | *yfbK* | | b2270 | | | | | 58 | | | 1.44 | | | |  | |  | | | | | hypothetical protein | |  |  |
|  | *yfdV* | | b2372 | | | | | 17 | | | 1.62 | | | |  | |  | | | | | putative receptor protein | |  |  |
|  | *yfhM* | | b2520 | | | | | 108 | | | 1.37 | | | |  | |  | | | | | hypothetical protein | |  |  |
|  | *yidK* | | b3679 | | | | | 116 | | | 1.36 | | | |  | |  | | | | | putative cotransporter | |  |  |
|  | *yieF* | | b3713 | | | | | 201 | | | 1.23 | | | |  | |  | | | | | hypothetical protein | |  |  |
|  | *yieH* | | b3715 | | | | |  | | |  | | | | 41 | | 1.87 | | | | | putative phosphatase | |  |  |
|  | *yjbP* | | b4055 | | | | |  | | |  | | | | 88 | | 1.40 | | | | | diadenosine tetraphosphatase | |  |  |
|  | *yjdA* | | b4109 | | | | | 60 | | | 1.44 | | | |  | |  | | | | | putative vimentin | |  |  |
|  | *ymdB* | | b1045 | | | | |  | | |  | | | | 7 | | 2.52 | | | | | putative polyprotein | |  |  |
|  | *ypfI* | | b2474 | | | | | 67 | | | 1.43 | | | |  | |  | | | | | hypothetical protein | |  |  |
|  | *yrbH* | | b3197 | | | | | 73 | | | 1.42 | | | |  | |  | | | | | putative isomerase | |  |  |
|  | *ytfL* | | b4218 | | | | | 77 | | | 1.42 | | | |  | |  | | | | | putative transport protein | |  |  |
| ***S: Function unknown*** | | | | | | | | | | | | | | | | | | | | | | | |  |  |
|  | *erfK* | b1990 | | | | |  | | | | | |  | 57 | | | | 1.68 | | | hypothetical protein | | | | |
|  | *ycaR* | | b0917 | | | | | 159 | | | 1.30 | | | |  | |  | | | | | hypothetical protein | |  |  |
|  | *yciF******** | | b1258 | | | | |  | | |  | | | | 27 | | 2.01 | | | | | YciF | |  |  |
| **No COG classification** | | | | | | | | | | | | | | | | | | | | | | | |  |  |
|  | *abgA* | | | b1338 | | | 101 | | | | | 1.38 | |  | | | | |  | | putative aminohydrolase | | | | |
|  | *aldB* | | b3588 | | | | | 63 | | | 1.43 | | | |  | |  | | | | | aldehyde dehydrogenase B | |  |  |
|  | *arsB* | | b3502 | | | | | 78 | | | 1.41 | | | |  | |  | | | | | arsenical pump membrane protein | |  |  |
|  | *b0816* | | b0816 | | | | |  | | |  | | | | 42 | | 1.86 | | | | | hypothetical protein | |  |  |
|  | *b2596* | | b2596 | | | | |  | | |  | | | | 11 | | 2.27 | | | | | hypothetical protein | |  |  |
|  | *fliO* | | b1947 | | | | |  | | |  | | | | 54 | | 1.73 | | | | | FliO | |  |  |
|  | *fruL* | | b0079 | | | | |  | | |  | | | | 36 | | 1.96 | | | | | fruR leader peptide | |  |  |
|  | *gapC_2* | | b1416 | | | | | 136 | | | 1.33 | | | |  | |  | | | | |  | |  |  |
|  | *gefL******** | | b0018 | | | | |  | | |  | | | | 24 | | 2.09 | | | | | Gef | |  |  |
|  | *insB_6* | | b3445 | | | | |  | | |  | | | | 28 | | 2.01 | | | | | IS1 protein InsB | |  |  |
|  | *kdgT* | | b3909 | | | | | 36 | | | 1.52 | | | |  | |  | | | | | 2-keto-3-deoxy-D-gluconate transport system | |  |  |
|  | *manZ* | | b1819 | | | | |  | | |  | | | | 99 | | 1.28 | | | | | PTS enzyme IID, mannose-specific | |  |  |
|  | *molR_2* | | b2116 | | | | | 149 | | | 1.30 | | | |  | |  | | | | |  | |  |  |
|  | *nanT* | | b3224 | | | | | 166 | | | 1.29 | | | |  | |  | | | | | sialic acid transporter | |  |  |
|  | *pinO* | | b3322 | | | | |  | | |  | | | | 8 | | 2.46 | | | | | PinO | |  |  |
|  | *pyrL* | | b4246 | | | | |  | | |  | | | | 64 | | 1.62 | | | | | pyrBI operon leader peptide | |  |  |
|  | *relF******** | | b1562 | | | | | 1 | | | 4.85 | | | | 1 | | 7.52 | | | | | RelF | |  |  |
|  | *rzpD******** | | b0556 | | | | | 18 | | | 1.62 | | | |  | |  | | | | | endopeptidase-like protein | |  |  |
|  | *smf_2 (f253)* | | b3286 | | | | | 45 | | | 1.49 | | | | 100 | | 1.28 | | | | |  | |  |  |
|  | *stfE******** | | b1157 | | | | |  | | |  | | | | 56 | | 1.71 | | | | | putative tail fiber protein | |  |  |
|  | *syd* | | | b2793 | | | 138 | | | | | 1.32 | |  | | | | |  | | Syd | | | | |
|  | *tus* | | b1610 | | | | |  | | |  | | | | 53 | | 1.73 | | | | | DNA-binding protein | |  |  |
|  | *wcaD* | | b2056 | | | | |  | | |  | | | | 14 | | 2.23 | | | | | putative colanic acid polymerase | |  |  |
|  | *yacH* | | b0117 | | | | |  | | |  | | | | 103 | | 1.21 | | | | | putative membrane protein | |  |  |
|  | *yafX******** | | b0248 | | | | | 33 | | | 1.55 | | | |  | |  | | | | | hypothetical protein | |  |  |
|  | *ybbD* | | b0500 | | | | | 49 | | | 1.48 | | | |  | |  | | | | | hypothetical protein | |  |  |
|  | *ybfP* | | b0689 | | | | |  | | |  | | | | 62 | | 1.63 | | | | | putative pectinase | |  |  |
|  | *ybhD* | | b0768 | | | | | 88 | | | 1.39 | | | |  | |  | | | | | putative transcriptional regulator LYSR-type | |  |  |
|  | *yccV* | | b0966 | | | | | 37 | | | 1.52 | | | |  | |  | | | | | hypothetical protein | |  |  |
|  | *yceP******** | | b1060 | | | | |  | | |  | | | | 6 | | 2.52 | | | | | hypothetical protein | |  |  |
|  | *ycfP* | | b1108 | | | | |  | | |  | | | | 15 | | 2.18 | | | | | hypothetical protein | |  |  |
|  | *ycgI* | | b1173 | | | | | 153 | | | 1.30 | | | |  | |  | | | | | hypothetical protein | |  |  |
|  | *yciC* | | b1255 | | | | | 175 | | | 1.27 | | | |  | |  | | | | | hypothetical protein | |  |  |
|  | *ycjM* | | b1309 | | | | | 145 | | | 1.31 | | | |  | |  | | | | | putative polysaccharide hydrolase | |  |  |
|  | *ydfN* | | b1547 | | | | | 173 | | | 1.27 | | | |  | |  | | | | | hypothetical protein | |  |  |
|  | *ydfZ******** | | b1541 | | | | | 9 | | | 1.79 | | | | 2 | | 2.77 | | | | | hypothetical protein | |  |  |
|  | *ydiN* | | b1691 | | | | | 193 | | | 1.25 | | | |  | |  | | | | | putative amino acid/amine transport protein | |  |  |
|  | *ydjM* | | b1728 | | | | |  | | |  | | | | 74 | | 1.51 | | | | | hypothetical protein | |  |  |
|  | *yfcT* | | b2337 | | | | | 111 | | | 1.37 | | | |  | |  | | | | | Fimbrial usher protein, C-terminal fragment | |  |  |
|  | *yfiE* | | b2577 | | | | | 165 | | | 1.29 | | | |  | |  | | | | | putative transcriptional regulator LYSR-type | |  |  |
|  | *yfjH* | | b2623 | | | | | 80 | | | 1.41 | | | |  | |  | | | | | putative histone | |  |  |
|  | *yfjW* | | b2642 | | | | |  | | |  | | | | 65 | | 1.61 | | | | | hypothetical protein | |  |  |
|  | *ygaX* | | b2680 | | | | |  | | |  | | | | 98 | | 1.28 | | | | |  | |  |  |
|  | *yghJ* | | b2974 | | | | | 41 | | | 1.50 | | | |  | |  | | | | | putative endoglucanase | |  |  |
|  | *yhbC* | | b3170 | | | | |  | | |  | | | | 63 | | 1.62 | | | | | hypothetical protein | |  |  |
|  | *yhdJ* | | b3262 | | | | | 177 | | | 1.26 | | | |  | |  | | | | | putative methyltransferase | |  |  |
|  | *yhdW* | | b3268 | | | | | 97 | | | 1.38 | | | |  | |  | | | | |  | |  |  |
|  | *yhdX* | | b3269 | | | | | 200 | | | 1.24 | | | |  | |  | | | | | putative transport system permease protein | |  |  |
|  | *yheB* | | b3338 | | | | | 103 | | | 1.37 | | | |  | |  | | | | | hypothetical protein | |  |  |
|  | *yhfL* | | b3369 | | | | |  | | |  | | | | 20 | | 2.12 | | | | | hypothetical protein | |  |  |
|  | *yhhM* | | b3467 | | | | | 195 | | | 1.24 | | | |  | |  | | | | | putative receptor | |  |  |
|  | *yhjN* | | b3532 | | | | | 55 | | | 1.45 | | | |  | |  | | | | | hypothetical protein | |  |  |
|  | *yhjU* | | b3538 | | | | | 85 | | | 1.40 | | | |  | |  | | | | | hypothetical protein | |  |  |
|  | *yiaF******** | | b3554 | | | | | 16 | | | 1.65 | | | |  | |  | | | | | hypothetical protein | |  |  |
|  | *yiaW* | | b3587 | | | | | 51 | | | 1.48 | | | |  | |  | | | | | hypothetical protein | |  |  |
|  | *yicJ* | | b3657 | | | | | 121 | | | 1.35 | | | |  | |  | | | | | putative permease | |  |  |
|  | *yidE* | | b3685 | | | | | 210 | | | 1.21 | | | |  | |  | | | | | putative transport protein | |  |  |
|  | *yieI* | | b3716 | | | | | 203 | | | 1.22 | | | |  | |  | | | | | hypothetical protein | |  |  |
|  | *yjiT* | | b4342 | | | | | 143 | | | 1.31 | | | |  | |  | | | | | hypothetical protein | |  |  |
|  | *ylcE******** | | b0563 | | | | |  | | |  | | | | 4 | | 2.55 | | | | | hypothetical protein | |  |  |
|  | *yliH******** | | b0836 | | | | | 3 | | | 2.47 | | | | 18 | | 2.15 | | | | | putative receptor | |  |  |
|  | *ynaA* | | b1368 | | | | | 66 | | | 1.43 | | | |  | |  | | | | | putative alpha helix protein | |  |  |
|  | *ypjC******** | | b2650 | | | | |  | | |  | | | | 33 | | 1.99 | | | | | hypothetical protein | |  |  |

**a.** Gene names according to *E.coli EcoCyc* database (www.ecocyc.org).

**b.** Gene names according to Blattner nomenclature (http://www.genome.wisc.edu/sequencing/k12.htm#gen).

**c.** Comparison of gene expression in MG1655 F’ + 55989*a* biofilm (C + P) versus gene expression in MG1655 F’ biofilm (C) expressed as ratio.

**d.** Comparison of gene expression in MG1655 F’ + 55989*a* biofilm (C + P) versus gene expression in MG1655 F’ + MG1655 F’ biofilm (C + C).

**e.** Rank position; 1 = most overexpressed gene in mixed biofilm (C+P) versus non-infected commensal (C) or self-infected (C + C) biofilm.

**f.** Function description according to COG annotation system used by the NCBI (http://www.ncbi.nlm.nih.gov/COG)

 :genes that are also overexpressed in response to colonization of commensal biofilm alone (C + C / C ; **see Table S2**).

*****:Genes chosen for further analysis.
