# Supplementary material for: Identification of Commensal Escherichia coli Genes Involved in Biofilm Resistance to Pathogen Colonization
Source: PLoS One. 2013 May 7;8(5):e61628. doi: 10.1371/journal.pone.0061628 (PMC3646849; doi:10.1371/journal.pone.0061628)
Supplement: Table S5 — Genes repressed upon colonization by exogenous pathogen (C+P). (DOCX) [file pone.0061628.s007.docx]

**Table S5:** Genes repressed upon colonization by exogenous pathogen (C+P).

| **Gene Name** | | | C + P /C^c^ | | C + P / **C + C^d^** | | **Function-description^f^** |
| --- | --- | --- | --- | --- | --- | --- | --- |
| **a** | | **b** | **Rank^e^** | **Ratio** | **Rank^e^** | **Ratio** |  |
| **Information storage and processing** | | | | | | | |
| ***J: Translation. ribosomal structure and biogenesis*** | | | | | | | |
|  | prfC | b4375 | 107 | 0.83 |  |  | peptide chain release factor RF-3 |
|  | rplK | b3983 | 58 | 0.70 | 28 | 0.74 | 50S ribosomal subunit protein L11 |
|  | rplP | b3313 |  |  | 57 | 0.84 | 50S ribosomal subunit protein L16 |
|  | rplY | b2185 | 78 | 0.73 |  |  | 50S ribosomal subunit protein L25 |
|  | rpmC | b3312 |  |  | 50 | 0.81 | 50S ribosomal subunit protein L29 |
|  | rpmJ | b3299 | 108 | 0.84 |  |  | 50S ribosomal subunit protein L36 |
|  | rpsC | b3314 |  |  | 60 | 0.85 | 30S ribosomal subunit protein S3 |
|  | yaeJ | b0191 | 105 | 0.82 |  |  | hypothetical protein |
|  | yciL | b1269 | 79 | 0.74 |  |  | hypothetical protein |
|  | yfiF | b2581 |  |  | 53 | 0.82 | hypothetical protein |
|  | yibK | b3606 |  |  | 37 | 0.77 | hypothetical protein |
| ***A : RNA processing and modification*** | | | | | | | |
|  | yhgJ | b3419 |  |  | 31 | 0.75 | RNA phosphage cyclase |
| *K: Transcription* | | | | | | | |
|  | iclR | b4018 |  |  | 44 | 0.79 | IclR |
|  | rpoC | b3988 |  |  | 61 | 0.85 | RNA polymerase beta prime subunit |
|  | ycjW | b1320 |  |  | 32 | 0.76 | putative LACI-type transcriptional regulator |
|  | yfeG | b2437 | 96 | 0.78 |  |  | putative ARAC-type regulatory protein |
|  | yfjR | b2634 | 34 | 0.64 |  |  | hypothetical protein |
|  | ygiP | b3060 | 82 | 0.74 |  |  | putative transcriptional regulator LYSR-type |
|  | yhcS | b3243 | 94 | 0.77 |  |  | quorum sensing regulator A |
|  | yhhY | b3441 | 1 | 0.30 | 15 | 0.66 | hypothetical protein |
| ***L: DNA replication, recombination and repair*** | | | | | | | |
|  | b4272 | b4272 | 76 | 0.73 | 48 | 0.81 | IS2 hypothetical protein |
|  | helD | b0962 | 98 | 0.78 |  |  | DNA helicase IV |
|  | intR | b1345 | 93 | 0.77 |  |  | putative transposase |
|  | rep | b3778 | 103 | 0.80 | 52 | 0.82 | rep helicase |
|  | yafM | b0228 | 10 | 0.57 |  |  | hypothetical protein |
| Cellular processes | | | | | | | |
| ***D: Cell cycle control, cell division, chromosome partitioning*** | | | | | | | |
|  | yefM | b2017 | 35 | 0.64 |  |  | hypothetical protein |
| ***O: Posttranslational modification, protein turnover. chaperones*** | | | | | | | |
|  | cbpA | b1000 | 5 | 0.51 |  |  | curved DNA-binding protein |
|  | hhoA | b3234 |  |  | 42 | 0.79 | serine endoprotease |
|  | hhoB | b3235 |  |  | 33 | 0.76 | protease |
|  | hypF | b2712 | 18 | 0.61 | 41 | 0.78 | transcriptional regulatory protein |
| ***M: Cell envelope biogenesis, outer membrane*** | | | | | | | |
|  | nlpC | b1708 | 20 | 0.61 |  |  | lipoprotein |
|  | rfbB | b2041 | 92 | 0.77 |  |  | dTDP-glucose 4,6 dehydratase |
|  | rlpB | b0641 | 97 | 0.78 |  |  | RlpB |
|  | spr | b2175 | 91 | 0.77 |  |  | putative lipoprotein |
|  | yohG | b2138 | 99 | 0.79 |  |  | putative channel/filament proteins |
| ***P: Inorganic ion transport and metabolism*** | | | | | | | |
|  | cysP | b2425 | 36 | 0.65 |  |  | thiosulfate binding protein |
|  | kdpA | b0698 |  |  | 4 | 0.56 | KdpA |
|  | modB | b0764 | 9 | 0.56 |  |  | molybdate transport permease protein |
|  | ycdN | b1016 | 2 | 0.40 |  |  | high-affinity iron permease |
|  | ychM | b1206 | 19 | 0.61 |  |  | hypothetical protein |
|  | yfeP | b2392 | 63 | 0.70 |  |  | putative transport system permease |
|  | yjbB | b4020 |  |  | 39 | 0.77 | putative alpha helix protein |
| ***T: Signal transduction mechanisms*** | | | | | | | |
|  | apaH | b0049 | 100 | 0.79 |  |  | diadenosine tetraphosphatase |
|  | chpB | b4225 | 21 | 0.62 |  |  | ChpB |
|  | chpR | b2783 | 14 | 0.60 |  |  | ChpR |
|  | chpS | b4224 | 52 | 0.69 |  |  | ChpS |
|  | yhjH | b3525 | 46 | 0.68 |  |  | hypothetical protein |
| ***U: Intracellular trafficking, secretion and vesicular transport*** | | | | | | | |
|  | yheI | b3331 | 66 | 0.71 |  |  | putative export protein I |
|  | yjaI | b4002 | 69 | 0.71 | 35 | 0.76 | hypothetical protein |
| **Metabolism** | | | | | | | |
| C: Energy production and conversion | | | | | | | |
|  | cydD | b0887 |  |  | 58 | 0.85 | ATP-binding component of cytochrome-related transport |
|  | hyaD | b0975 | 68 | 0.71 | 49 | 0.81 | HyaD |
|  | yfhL | b2562 |  |  | 5 | 0.58 | hypothetical protein |
|  | yhaA | b3115 | 87 | 0.75 |  |  | putative kinase |
|  | yibA | b3594 | 74 | 0.73 | 34 | 0.76 | hypothetical protein |
| ***G: Carbohydrate transport and metabolism*** | | | | | | | |
|  | agaW | b3134 | 104 | 0.81 |  |  | PTS system N-acetylgalactosameine-specific IIC component 2 |
|  | exuT | b3093 | 83 | 0.74 |  |  | ExuT |
|  | frwB | b3950 |  |  | 43 | 0.79 | PTS system fructose-like IIB component 1 |
|  | gatY | b2096 | 84 | 0.75 | 38 | 0.77 | tagatose-bisphosphate aldolase 1 |
|  | rbsD | b3748 | 67 | 0.71 |  |  | RbsD |
|  | ybjJ | b0845 | 61 | 0.70 | 46 | 0.80 | putative DEOR-type transcriptional regulator |
|  | ydeF | b1534 |  |  | 40 | 0.78 | putative transport protein |
|  | yhfP | b3373 | 102 | 0.80 |  |  | hypothetical protein |
| ***E: Amino acid transport and metabolism*** | | | | | | | |
|  | gloA | b1651 |  |  | 20 | 0.68 | lactoylglutathione lyase |
|  | gltL | b0652 | 86 | 0.75 | 25 | 0.73 | GltL |
|  | goaG | b1302 |  |  | 36 | 0.77 | 4-aminobutyrate aminotransferase |
|  | hisF | b2025 | 37 | 0.65 |  |  | imidazole glycerol phosphate synthase holoenzyme |
|  | metC | b3008 | 47 | 0.68 |  |  | cystathionine beta-lyase |
|  | metF | b3941 |  |  | 29 | 0.75 | 5,10-methylenetetrahydrofolate reductase |
|  | proV | b2677 |  |  | 11 | 0.65 | ATP-binding component of transport system for glycine, betaine and proline |
|  | serA | b2913 | 32 | 0.63 |  |  | D-3-phosphoglycerate dehydrogenase |
|  | speB | b2937 | 26 | 0.62 |  |  | agmatinase |
|  | ygeY | b2872 | 70 | 0.71 |  |  | putative deacetylase |
|  | yggP | b2931 | 24 | 0.62 |  |  | putative oxidoreductase |
|  | yifK | b3795 | 59 | 0.70 |  |  | putative amino acid/amine transport protein |
| ***F: Nucleotide transport and metabolism*** | | | | | | | |
|  | adk | b0474 | 44 | 0.67 |  |  | Adk |
|  | nrdI | b2674 | 54 | 0.69 |  |  | hypothetical protein |
|  | ushA | b0480 | 27 | 0.62 | 21 | 0.68 | UDP-sugar hydrolase (5'-nucleotidase) |
|  | yeiK | b2162 |  |  | 51 | 0.81 | hypothetical protein |
| ***H: Coenzyme metabolism*** | | | | | | | |
|  | hemX | b3803 | 109 | 0.89 |  |  | uroporphyrinogen III methylase |
|  | ygdL | b2812 | 30 | 0.63 | 16 | 0.66 | hypothetical protein |
| ***I: Lipid metabolism*** | | | | | | | |
|  | psd | b4160 | 22 | 0.62 |  |  | phosphatidylserine decarboxylase |
|  | yciA | b1253 | 42 | 0.67 |  |  | hypothetical protein |
|  | ***Q: Secondary metabolite biosynthesis, transport and catabolism*** | | | | | | |
|  | yecD | b1867 | 39 | 0.66 |  |  | hypothetical protein |
| Poorly characterized | | | | | | | |
| ***R: General function prediction only*** | | | | | | | |
|  | b0165  (f43) | b0165 | 15 | 0.60 |  |  | hypothetical protein |
|  | bioH | b3412 | 64 | 0.71 |  |  | BioH |
|  | ychK | b1234 | 65 | 0.71 | 23 | 0.71 | hypothetical protein |
|  | yfiO | b2595 | 81 | 0.74 |  |  | hypothetical protein |
|  | ygfQ | b2885 | 77 | 0.73 |  |  | putative oxidoreductase |
|  | yieM | b3745 | 80 | 0.74 | 59 | 0.85 | hypothetical protein |
|  | yjgR | b4263 | 17 | 0.60 | 30 | 0.75 | hypothetical protein |
|  | yoaE | b1816 | 13 | 0.59 | 9 | 0.63 | putative transport protein |
| ***S: Function unknown*** | | | | | | | |
|  | yaeQ | b0190 | 31 | 0.63 | 26 | 0.73 | hypothetical protein |
|  | ydbL | b1383 | 3 | 0.40 |  |  | hypothetical protein |
|  | ydiC | b1684 | 56 | 0.70 |  |  | hypothetical protein |
|  | ydjC | b1733 |  |  | 18 | 0.67 | hypothetical protein |
|  | yeeA | b2008 | 62 | 0.70 |  |  | hypothetical protein |
|  | ygiH | b3059 | 29 | 0.63 |  |  | hypothetical protein |
|  | ygjQ | b3086 | 50 | 0.68 | 19 | 0.67 | hypothetical protein |
|  | yhbP | b3154 | 90 | 0.76 | 12 | 0.65 | hypothetical protein |
|  | yhdT | b3257 | 25 | 0.62 |  |  | hypothetical protein |
|  | yhgI | b3414 |  |  | 45 | 0.79 | hypothetical protein |
|  | yhgL | b3421 |  |  | 56 | 0.83 | hypothetical protein |
|  | yjbA | b4030 | 23 | 0.62 |  |  | hypothetical protein |
|  | yjbR | b4057 | 95 | 0.78 |  |  | hypothetical protein |
|  | yjiH | b4330 |  |  | 14 | 0.66 | hypothetical protein |
|  | yneE | b1520 |  |  | 8 | 0.63 | hypothetical protein |
|  | yqjE | b3099 | 75 | 0.73 | 47 | 0.80 | hypothetical protein |
| **No COG classification** | | | | | | | |
|  | appA | b0980 | 45 | 0.68 |  |  | phosphoanhydride phosphorylase |
|  | b0667 | b0667 | 51 | 0.68 |  |  |  |
|  | ilvL | b3766 | 49 | 0.68 |  |  | ilvGEDA operon leader peptide |
|  | o149 | b3677 | 7 | 0.52 |  |  | hypothetical protein |
|  | pssA | b2585 |  |  | 24 | 0.72 | phosphatidylserine synthase |
|  | tfaD | b0561 |  |  | 27 | 0.73 | hypothetical protein |
|  | trpL | b1265 | 4 | 0.43 |  |  | trp operon leader peptide |
|  | wcaM | b2043 | 101 | 0.80 |  |  | hypothetical protein |
|  | yacC | b0122 | 106 | 0.83 |  |  | hypothetical protein |
|  | yajD | b0410 | 41 | 0.66 |  |  | hypothetical protein |
|  | ybcC | b0539 |  |  | 7 | 0.60 | putative exonuclease |
|  | ybcU | b0557 |  |  | 6 | 0.58 | Bor-like protein |
|  | ydaC | b1347 | 6 | 0.51 |  |  | hypothetical protein |
|  | yddG | b1473 | 73 | 0.72 |  |  | hypothetical protein |
|  | ydhW | b1672 | 55 | 0.69 |  |  | hypothetical protein |
|  | yfbN | b2273 | 89 | 0.76 |  |  | hypothetical protein |
|  | yfcU | b2338 | 71 | 0.72 |  |  |  |
|  | yfdY | b2377 | 43 | 0.67 |  |  | hypothetical protein |
|  | ygbF | b2754 | 53 | 0.69 |  |  | hypothetical protein |
|  | ygdB | b2824 | 11 | 0.58 | 2 | 0.54 | hypothetical protein |
|  | ygfI | b2921 | 88 | 0.76 |  |  | putative transcriptional regulator LYSR-type |
|  | yghR | b2984 | 72 | 0.72 |  |  | hypothetical protein |
|  | yghW | b2998 | 85 | 0.75 |  |  | hypothetical protein |
|  | ygiQ | b3016 | 60 | 0.70 |  |  |  |
|  | ygjV | b3090 | 33 | 0.64 | 3 | 0.55 | hypothetical protein |
|  | yhiM | b3491 |  |  | 54 | 0.83 | hypothetical protein |
|  | yibL | b3602 |  |  | 55 | 0.83 | hypothetical protein |
|  | yibQ | b3614 | 57 | 0.70 | 17 | 0.66 | hypothetical protein |
|  | yieN | b3746 | 28 | 0.62 |  |  | putative 2-component regulator |
|  | yigI | b3820 |  |  | 22 | 0.70 | hypothetical protein |
|  | yijI | b3948 | 40 | 0.66 |  |  | hypothetical protein |
|  | yjaH | b4001 | 12 | 0.58 | 10 | 0.64 | hypothetical protein |
|  | yjbD | b4023 | 38 | 0.66 |  |  | hypothetical protein |
|  | yjis | b4341 | 16 | 0.60 | 13 | 0.65 | hypothetical protein |
|  | ynaJ | b1332 | 8 | 0.55 |  |  | hypothetical protein |
|  | yoaC | b1810 |  |  | 1 | 0.41 | hypothetical protein |
|  | yqhE | b3012 | 48 | 0.68 |  |  | hypothetical protein |

**a.** Gene names according to *E. coli* Colibri database (http://genolist.pasteur.fr/Colibri/).

**b.** Gene names according to Blattner nomenclature (http://www.genome.wisc.edu/sequencing/k12.htm#gen).

**c.** Comparison of gene expression in MG1655 F’ + 55989*a* biofilm (C + P) versus gene expression in MG1655 F’ biofilm (C) expressed as ratio.

**d.** Comparison of gene expression in MG1655 F’ + 55989*a* biofilm (C + P) versus gene expression in MG1655 F’ + MG1655 F’ biofilm (C + C).

**e.** Rank position; 1 = most underexpressed gene in mixed biofilm (C+P) versus non-infected commensal (C) or self-infected (C + C) biofilm.

**f.** Function description according to COG functional categories annotation system used by the NCBI (<http://www.ncbi.nlm.nih.gov/COG>).

Genes that are also repressed in response to colonization of commensal biofilm alone (C + C / C **; see Table S3**).
