# Supplementary material for: Identification of Commensal Escherichia coli Genes Involved in Biofilm Resistance to Pathogen Colonization
Source: PLoS One. 2013 May 7;8(5):e61628. doi: 10.1371/journal.pone.0061628 (PMC3646849; doi:10.1371/journal.pone.0061628)
Supplement: Table S6 — Primers used in this study. (DOCX) [file pone.0061628.s008.docx]

**Table S6: Primers used in this study**

| **Primer name** | **Primer sequence** |
| --- | --- |
| **Deletion mutants** | |
| relF-500-5 | tcgctgacaatgaacgcttg |
| relF-ext5 | tagcattaacctgcgtattg |
| relF-GBL-3 | GATTTTGAGACACAACGTGGCTTTCCATAGCCTGCTTCTCCTGTCA |
| relF-500-3 | gtgttactcagcgggagttc |
| relF-ext3 | gacttgaatgcgtgagaata |
| relF-GBLnp-5 | TGCTCGATGAGTTTTTCTAAgagacccggcgggggagaaatc |
| kduI500-5 | atgaagcgtttgctgcacagg |
| kduI-ext5 | ttgcgccggtgtatgcgacc |
| kduI-GBL3 | GATTTTGAGACACAACGTGGCTTTCCATATCAACCTCCGATAATCAGTG |
| kduI-500-3 | gcggtataagaaggcacac |
| kdui-ext3 | gcattaacattaatgttgtg |
| kduI-GBL5 | TGCTCGATGAGTTTTTCTAAttgtgggcataaacgaataagg |
| yliH-500-5 | cagcactttcgggtggacttc |
| yliH-ext5 | caggaatgggttgtgtttcg |
| yliH-GBL-3 | GATTTTGAGACACAACGTGGCTTTCCATACTTCGATCCTCCTCTTCCCA |
| yliH-500-3 | ggaagtcggtcacttttgag |
| yliH-ext3 | cagagcaataaaaagataac |
| yliH-GBLnp-5 | TGCTCGATGAGTTTTTCTAAaaactaagggggagaaagcgtc |
| ydfZ-500-5 | atgatttttttgaacttgatg |
| ydfZ-ext5 | caggccattgagtgcgcggtg |
| ydfZ-GBL-3 | GATTTTGAGACACAACGTGGCTTTCCATaggtgttttctcctttctgatt |
| ydfZ-500-3 | cgttgatttgcaagattacgc |
| ydfZ-ext3 | gatatcagcacattaatgac |
| ydfZ-GBLnp-5 | TGCTCGATGAGTTTTTCTAAgcgtgtgaatgccgccgatgg |
| ylcE.500-5 | gaaggtaagacatggaaatg |
| ylcE.ext-5 | catcgtcagttaaggcctgc |
| ylcE.GBL-3 | GATTTTGAGACACAACGTGGCTTTCTTCCTGGATGTAGAACTGTGTGGT |
| ylcE.500-3 | aaccaattaaatacacaatc |
| ylcE.ext-3 | ctacggagcaaacataatcc |
| ylcE.GBLnp-5 | TGCTCGATGAGTTTTTCTAAAtaaacagtatgtatatcatagg |
| yciF.500-5 | gtcaacttacgtcatttttcc |
| yciF.GBL-3 | GATTTTGAGACACAACGTGGCTTTCCATatttttctccagtgaaatc |
| yciF.500-3 | ctacgccatcagtttcagagc |
| yciF.GBLnp-5 | TGCTCGATGAGTTTTTCTAAaatatgaattttaacttttagtc |
| yciF.ext-5 | tttaatgaagccggaaaaatc |
| yciF.ext-3 | ggatatcgtttttaattccg |
| cspF.500-5 | cagtaatgatgacatttgc |
| cspF.ext-5 | ctgtccgtatctctactcag |
| cspF.GBL-3 | GATTTTGAGACACAACGTGGCTTTCCATaaaaattccttaatactgat |
| cspF.500-3 | gtataaggggtatgcggacg |
| cspF.ext-3 | accgaccacgctggcacgac |
| cspF.GBLnp-5 | TGCTCGATGAGTTTTTCTAAgctatattaaagctttaatttc |
| ypjC.500-5 | gtaattaacgataaagccag |
| ypjC.GBL-3 | GATTTTGAGACACAACGTGGCTTTCCATtgtctattcctcaattaatg |
| ypjC.ext-5 | tggaaaaaatgcctgctatg |
| ypjC.500-3 | taatgttaattttggctatc |
| ypjC.GBLnp-5 | TGCTCGATGAGTTTTTCTAAaaccttttcgataatataag |
| ypjC.ext-3 | gttctgtccagaaaagaagc |
| yliE.500-5 | ccgttttgttgcgtcatatc |
| yliE.GBL-3 | GATTTTGAGACACAACGTGGCTTTCCATgttaatacatttaacaaaat |
| yliE.500-3 | ttttgacagtaaagaaaaac |
| yliE.GBLnp-5 | TGCTCGATGAGTTTTTCTAAtttacgtatgtccagaatcaataag |
| yliE.ext-5 | cagtcgccattttttccatc |
| yliE.ext-3 | tttagcattatattcatcac |
| sppA.500-5 | caactgctgccatttgcatc |
| sppA.GBL-3 | GATTTTGAGACACAACGTGGCTTTCCATgtattctcccaacttaagg |
| sppA.500-3 | gcatgaacgacagcgcagag |
| sppA.GBLnp-5 | TGCTCGATGAGTTTTTCTAAgtcttgtactgagtggccg |
| sppA.ext-5 | ctgctcacggcaaccgaatg |
| sppA.ext-3 | tccgtcagagcgtaactcag |
| agaI.500-5 | ttttctggtttactttgttg |
| agaI.GBL-3 | GATTTTGAGACACAACGTGGCTTTCCATttacaaaatgccgaatg |
| agaI.500-3 | gagtcattgatttcacaagc |
| agaI.GBLnp-5 | TGCTCGATGAGTTTTTCTAAccagatgaaaaatttgctc |
| agaI.ext-5 | gcgacaccattaaaggcctc |
| agaI.ext-3 | ctgttcggcattatagtgac |
| rcsA.500-5 | gacactgaatctggcattag |
| rcsA.GBL-3 | GATTTTGAGACACAACGTGGCTTTCCATggcataccctcactcaatg |
| rcsA.500-3 | tcattgatcgccgccagcag |
| rcsA.GBLnp-5 | TGCTCGATGAGTTTTTCTAAcacattctgactggtggtttc |
| rcsA.ext-5 | tcaccaaagcagggagtttg |
| rcsA.ext-3 | atcgacgctaacgtgttcag |
| yjcR.500-5 | attaatgtcactcgcttttg |
| yjcR.GBL-3 | GATTTTGAGACACAACGTGGCTTTCCATgagcgcgtctcaaatag |
| yjcR.500-3 | ggccaacaacgatacaccac |
| yjcR.GBLnp-5 | TGCTCGATGAGTTTTTCTAAtgagcgcgctcaactccc |
| yjcR.ext-5 | cgaaactggaagaaattatc |
| yjcR.ext-3 | gcacgactgggaaaccacag |
| stfE.500-5 | attgttctggtgtaagtttg |
| stfE.GBL-3 | GATTTTGAGACACAACGTGGCTTTCCATgtttatgaaaatgaagaaataac |
| stfE.500-3 | tggttgtttctgcctgcgtc |
| stfE.GBLnp-5 | TGCTCGATGAGTTTTTCTAAtgcataaagcaatattaaatag |
| stfE.ext-5 | caccttctggcgaggagttc |
| stfE.ext-3 | ttgccgtcaccgcgtcaatg |
| yiaF.500-5 | tccaggctgctcaacagctc |
| yiaF.GBL-3 | GATTTTGAGACACAACGTGGCTTTCCATtttcttactcctttcac |
| yiaF.500-3 | ccaccataaagaagcagaag |
| yiaF.GBLnp-5 | TGCTCGATGAGTTTTTCTAAAagagtaaaagcccgagcg |
| yiaF.ext-5 | tttcgtaactgctcaatttc |
| yiaF.ext-3 | gtgcgcactttggcttcaac |
| rzpD.500-5 | ggagcaattgctatagcatc |
| rzpD.GBL-3 | GATTTTGAGACACAACGTGGCTTTCCATtgttgcccccacaaacagac |
| rzpD.500-3 | gttccccgtcagggctgtgg |
| rzpD.GBLnp-5 | TGCTCGATGAGTTTTTCTAAagctgaccatatcgatgggc |
| rzpD.ext-5 | acctgacaaacctttatttc |
| rzpD.ext-3 | aatacatgtacgcgctaaac |
| yaeT.500-5 | tattgagccgaaagtcattc |
| yaeT.GBL-3 | GATTTTGAGACACAACGTGGCTTTCCATcgttattatgcgttcttc |
| yaeT.500-3 | caacggatttcacagcagtc |
| yaeT.GBLnp-5 | TGCTCGATGAGTTTTTCTAAgtgttctccacaaaggaatg |
| yaeT.ext-5 | attaatcccggagagtaaac |
| yaeT.ext-3 | taagcaacggcgtttgcatc |
| ycbQ.500-5 | tgcagattccagtgataaac |
| ycbQ.GBL-3 | GATTTTGAGACACAACGTGGCTTTCCATcgttatcatcctgatctc |
| ycbQ.500-3 | ggcaatcttcagggtatttc |
| ycbQ.GBLnp-5 | TGCTCGATGAGTTTTTCTAAtagaactcattaattgttttatta |
| ycbQ.ext-5 | gtcttgagtgccggactatc |
| ycbQ.ext-3 | gcctaagggatacgctaaac |
| yafX.500-5 | ccttcactgcggaccggaag |
| yafX.GBL-3 | GATTTTGAGACACAACGTGGCTTTCCATgggtatgtctccgtcaataaa |
| yafX.500-3 | cctcaaagcagaccgtgttc |
| yafX.GBLnp-5 | TGCTCGATGAGTTTTTCTAAccgaaggagcaacagatgaaac |
| yafX.ext-5 | ttgacttcatcagcaatatc |
| yafX.ext-3 | gtatatcgtcattgccctgc |
| yjiY.500-5 | tgaaattttctttgtcattc |
| yjiY.GBL-3 | GATTTTGAGACACAACGTGGCTTTCCATagtaaaacctggcat |
| yjiY.500-3 | ccagctcgttggagcgcgaac |
| yjiY.GBLnp-5 | TGCTCGATGAGTTTTTCTAAccgtgtttagccccgcttc |
| yjiY.ext-5 | ctgccattcagcgtggattg |
| yjiY.ext-3 | ggtgcattcaatgaccagac |
| yiaV.500-5 | actatttcaatatgataaac |
| yiaV.GBL-3 | GATTTTGAGACACAACGTGGCTTTCCATaagtattactcggcag |
| yiaV.500-3 | cagttttattacccaatatg |
| yiaV.GBLnp-5 | TGCTCGATGAGTTTTTCTAAtcttcgctttccgccgcttg |
| yiaV.ext-5 | ctgtggatgttttactggag |
| yiaV.ext-3 | atgattgtgatgttttaatc |
| YceP.A1.500-5 | gCgAAAACTTCTCCATTgCC |
| YcePA2GBL-3 | gATTTTgAgACACAACgTggCTTTCATCATggCCCCCTAATTCg |
| YceP.B1.500-3 | CAgCgggCCATAATCCCTTg |
| YcePB2GBL-5 | CTTCACgAggCAgACCTCAgCgCCTAACATgACATgACCATCC |
| YceP.ext-5 | CCAgTATATTCAACAggggg |
| YcePext-3 | CTTCgCCAgTTggATCCAgg |
| **Overexpression mutants** | |
| ypjC-PcL-L3 | GTGAGAATTACTAACTTGAGCGAActacatggctatttttaatgttat |
| ypjC-PcL-500-3 | AGCGCATTCAAAAAACTGATCGG |
| ypjC-PcL-L5 | CGGTGATAATGGTTGCATGTACTAATGacattaattgaggaatagacaATG |
| ypjC-PcL-ext-3 | aagtaaaccaacatgaaaatc |
| yliE-PcL-L3 | GTGAGAATTACTAACTTGAGCGAAactatctgaacgctgtgccgctgc |
| yliE-PcL-500-3 | AGTCATCTGAAGCCACCGACC |
| yliE-PcL-L5 | CGGTGATAATGGTTGCATGTACTAATGtattttgttaaatgtattaacATG |
| yliE-PcL-ext-3 | AAAAGAGATACATTTTTTACC |
| rcsA-PcL-L3 | GTGAGAATTACTAACTTGAGCGAAtaacgataattccccttacctgaa |
| rcsA-PcL-500-3 | TTTGGTCAGAGATTTGAATGG |
| rcsA-PcL-L5 | CGGTGATAATGGTTGCATGTACTAATGcgcattgagtgagggtatgccATG |
| rcsA-PcL-ext-3 | ATTATGCGTTTTGATCTTACG |
| yiaF-PcL-L3 | GTGAGAATTACTAACTTGAGCGAAaaaaagcgcacaacgacacacgc |
| yiaF-PcL-500-3 | GTGAATGCCTGATCAAAGACC |
| yiaF-PcL-L5 | CGGTGATAATGGTTGCATGTACTAATGttgggtgaaaggagtaagaaaATG |
| yiaF-PcL-ext3 | TTTGTGCCGCCGGAATCAACG |
| stfE-PcL-L3 | GTGAGAATTACTAACTTGAGCGAAgatttaacatgcttattggctatg |
| stfE-PcL-500-3 | GATTGTTTATATGTTTTAAAGG |
| stfE-PcL-L5 | CGGTGATAATGGTTGCATGTACTAATGcccctggtaaaagcaggaatgATG |
| stfE-PcL-ext3 | ATAGTTAAATGCAATATTACG |
| **RT-PCR experiments** | |
| YceP-RT-5 | GCTCGTAGGGTGGGACATCA |
| YceP-RT-3 | CGAAATAAATTGTCTGGCAACATC |
| YiaF-RT-5 | ATTCGCGTACCGCAGGATTA |
| YiaF-RT-3 | CCGGTTTCAAATCATCGCTC |
| RscA-RT-5 | GCTTGTGATTCACAGCGCC |
| RscA-RT-3 | TCAAAATGAAATTGGCAATTGC |
| YliH-RT-5 | GAATCGATCTGCTGAACCGG |
| YliH-RT-3 | GATTTATCGTGCAGTTCGCG |
| GyrEc-RT-5 | GACGGTATTGGCGTCGAAGT |
| GyrEc-RT-3 | CCATGTAGGCGTTCAGGGTG |
| StfE-SG-5 | TGAAGAATACCCGGAACTGGC |
| StfE-SG-3 | AGCATGATCCTCCGTTGCA |
| YciF-SG-5 | CACTGGCAAAACTCGCAAGA |
| YciF-SG-3 | GCCACACATTTCATGCGCT |
| YliE-SG-5 | ATTGATGACTTTGGCACCGG |
| YliE-SG-3 | GGTTATAGCGTGCCAGGTCG |
